# Symptoms of Depression, Eating Disorders, and Binge Eating in Adolescents With Obesity: The Fast Track to Health Randomized Clinical Trial

**DOI:** 10.1001/jamapediatrics.2024.2851

**Published:** 2024-08-26

**Authors:** Hiba Jebeile, Louise A. Baur, Cathy Kwok, Shirley Alexander, Justin Brown, Clare E. Collins, Christopher T. Cowell, Kaitlin Day, Sarah P. Garnett, Megan L. Gow, Alicia M. Grunseit, Maddison Henderson, Eve T. House, Mary-Kate Inkster, Sarah Lang, Susan J. Paxton, Helen Truby, Krista A. Varady, Natalie B. Lister

**Affiliations:** 1 The University of Sydney, Sydney Medical School, Westmead, New South Wales, Australia; 2 The Children’s Hospital at Westmead, Institute of Endocrinology and Diabetes, Westmead, New South Wales, Australia; 3 The Children’s Hospital at Westmead, Weight Management Services, Westmead, New South Wales, Australia; 4 Monash Children’s Hospital, Department of Paediatric Endocrinology and Diabetes, Clayton, Victoria, Australia; 5 Monash University, Department of Paediatrics, Clayton, Victoria, Australia; 6 University of Newcastle, School of Health Sciences, College of Health, Medicine and Wellbeing, Callaghan, New South Wales, Australia; 7 Food and Nutrition Research Program, Hunter Medical research Institute, New Lambton Heights, New South Wales, Australia; 8 Kids Research, The Children’s Hospital at Westmead, Westmead, New South Wales, Australia; 9 Monash University, Nutrition, Dietetics & Food, Melbourne, Victoria, Australia; 10 School of Agriculture, Food and Ecosystem Sciences, University of Melbourne, Melbourne, Victoria, Australia; 11 The Children’s Hospital at Westmead, Nutrition and Dietetics, Westmead, New South Wales, Australia; 12 La Trobe University, School of Psychology and Public Health, Melbourne, Victoria, Australia; 13 School of Primary and Allied Health Care, Monash University, Melbourne, Victoria, Australia; 14 School of Human Movement and Nutrition Sciences, University of Queensland, Brisbane, Queensland, Australia; 15 University of Illinois, Department of Kinesiology and Nutrition, Chicago

## Abstract

**Importance:**

Depression and eating disorders are heightened for adolescents with obesity. Clinical reviews alongside self-report questionnaires are important to ensure appropriate intervention.

**Objective:**

To evaluate changes in self-report symptoms of depression, eating disorders, and binge eating in adolescents with obesity during the Fast Track to Health trial.

**Design, Setting, and Participants:**

This was a randomized clinical trial conducted from 2018 to 2023. It was a multisite trial conducted at children’s hospitals in Sydney, New South Wales, and Melbourne, Victoria, Australia, and included adolescents (13-17 years) with obesity (defined as adult equivalent body mass index ≥30; calculated as weight in kilograms divided by height in meters squared) and 1 or more related complications.

**Interventions:**

Duration was 52 weeks including a very low energy diet for 4 weeks followed by intermittent energy restriction (IER) or continuous energy restriction (CER).

**Main Outcomes and Measures:**

Self-report symptoms of depression (Center for Epidemiologic Studies Depression Scale–Revised 10-Item Version for Adolescents [CESDR-10]; scores 0-30), eating disorders (Eating Disorder Examination Questionnaire [EDE-Q]; scores 0-6), and binge eating (Binge Eating Scale [BES]; scores 0-46) were assessed. Adolescents were screened for depression and eating disorders (weeks 0, 4, 16, and 52) and monitored for the onset of new symptoms of disordered eating during dietetic consults.

**Results:**

Of 141 adolescents (median [IQR] age, 14.8 [12.9-17.9] years; 71 male [50.4%]) enrolled, median baseline EDE-Q score was 2.28 (IQR, 1.43-3.14), median baseline CESDR-10 score was 9.00 (IQR, 4.00-14.50), and median baseline BES score was 11.00 (IQR, 5.00-17.00). There were no differences between groups for change in CESDR-10 (mean difference at week 52, 0.75; 95% CI, −1.86 to 3.37), EDE-Q (mean difference at week 52, 0.02; 95% CI, −0.41 to 0.45), or BES (mean difference at week 52, −2.91; 95% CI, −5.87 to 0.05). The within-group reductions at week 4 were maintained at week 52, for CESDR-10 and EDE-Q, indicating reduced symptoms of depression and eating disorders. Within-group reductions on the BES were maintained in the IER group only. Seventeen adolescents (12.1%) required support or referral for depression and/or disordered eating, including 7 (5%; 5 IER, 2 CER) adolescents who experienced the onset or reemergence of symptoms during the intervention.

**Conclusions and Relevance:**

Results suggest that many treatment-seeking adolescents with obesity self-reported symptoms of depression and eating disorders. Although symptoms reduced for most, some required additional support. Obesity treatment is an opportune time to screen and monitor for depression and disordered eating.

**Trial Registration:**

Australian New Zealand Clinical Trials Registry: ACTRN12617001630303

## Key Points

**Question:**

How do self-reported symptoms of depression, eating disorders, and binge eating change for adolescents with obesity during an intensive behavioral intervention?

**Findings:**

In this randomized clinical trial of 141 adolescents, symptoms of depression, eating disorders, and binge eating reduced after 4 weeks of a very low energy diet, and this reduction was maintained to 52 weeks after transition to intermittent or continuous energy restriction. A subset of adolescents required additional support for depression and/or disordered eating.

**Meaning:**

Results suggest that obesity treatment interventions may have a dual role of improving physiological and psychosocial health; screening and monitoring for depression and disordered eating are important to facilitate early intervention.

## Teaser

This randomized clinical trial investigates how self-reported symptoms of depression, eating disorders, and binge eating change for adolescents with obesity during an intensive behavioral intervention.

## Introduction

Adolescents with obesity are vulnerable to impaired psychosocial health.^1^ Through this life stage, symptoms of depression and eating disorders increase, and adolescents with obesity are at higher risk compared with peers with lower weight.^2^ Depression and disordered eating symptoms were exacerbated during the COVID-19 pandemic in adolescents, and obesity prevalence increased.^6^ Youth with obesity and disordered eating are likely to experience exacerbated physical and psychological health issues.^11^ It is important to understand the proportion of adolescents with obesity who are seeking treatment and experiencing these symptoms and effects of obesity treatment.^12^

Intensive behavioral lifestyle intervention is first-line treatment for adolescents seeking treatment for obesity and associated complications.^13^ Systematic reviews have examined change in symptoms of depression and eating disorders after structured pediatric obesity interventions.^16^ Meta-analyses indicated a mean reduction in symptoms of depression, binge eating, and shape and weight concerns, with no change in eating concerns or global risk after the intervention. All outcomes were reduced at a follow-up of 14 weeks to 6 years from baseline.^16^ Similarly, reductions in binge eating and loss of control eating have been reported after behavioral weight management, pharmacotherapy, and bariatric surgery for adolescent obesity.^18^ However, most included studies involved moderate dietary interventions, providing nutrition education alone or with a moderate continuous energy restriction. These findings cannot be extrapolated to more prescriptive dietary interventions or higher levels of energy restriction (eg, very low energy diets [VLEDs] or intermittent energy restriction [IER]),^22^ as more restrictive dieting practices have been associated with disordered eating in adolescent community samples.^23^ These approaches are recommended for use in adolescents with severe obesity and/or concurrent complications, warranting further investigation.^24^

It is important to understand the effect of obesity treatment on symptoms of depression and disordered eating and how these change over time. This knowledge can be used to inform health services, increasing awareness and understanding of these issues by clinicians, and inform psychological care needs. We aimed to assess change in self-report symptoms of depression, eating disorders, and binge eating during the Fast Track to Health trial.

## Methods

Fast Track to Health was a parallel, multicenter, randomized clinical trial conducted in Sydney, New South Wales, and Melbourne, Victoria, Australia, between January 31, 2018, and March 31, 2023. The protocol has been published,^26^ was approved by The Sydney Children’s Hospitals Network Human Research Ethics Committee (HREC/17/SCHN/164), and was reported according to the Consolidated Standards of Reporting Trials (CONSORT) reporting guidelines. The trial protocol, amendments, and statistical analysis plan are available in Supplement 1, Supplement 2, and Supplement 3, respectively. The primary outcome was change in body mass index (BMI) *z* score at 52 weeks, reported separately.^27^

### Participants

Eligible adolescents were aged 13 to 17 years with obesity (defined as the adult equivalent BMI ≥30; calculated as weight in kilograms divided by height in meters squared)^28^ and 1 or more cardiometabolic complications (eg, insulin resistance, hypertension).^26^ Agreement to participate was obtained from adolescents and written consent from parents/carers. Information on ethnicity was collected as per standard clinical practice for clinical trials.

### Intervention

Fast Track to Health was a 52-week intensive behavioral intervention comparing 2 dietary approaches, IER and continuous energy restriction (CER).^26^ In brief, the trial included 3 phases: (1) weeks 0 to 4, VLED (approximately 800 kcal per day); (2) weeks 5 to 16, participants transitioned to IER or CER; and (3) weeks 17 to 52, continued intervention and/or maintenance with reduced support. IER involved 3 energy-restricted days per week (approximately 600-700 kcal per day), and 4 days per week of healthy eating without energy restriction. CER involved tailored energy prescription based on age (13-14 years, 1430-1670 kcal per day; 15-17 years, 1670-1900 kcal per day).

### Screening and Monitoring

**Figure 1.**
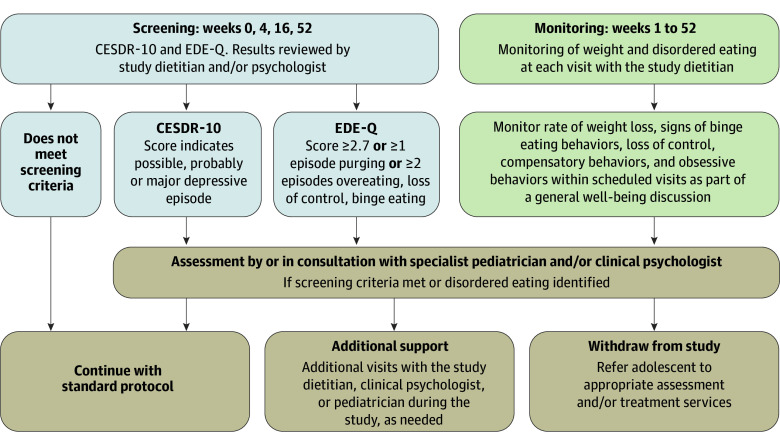
Depression and Eating Disorder Screening and Monitoring Protocol CESDR-10 indicates Center for Epidemiologic Studies Depression Scale–Revised 10-Item Version for Adolescents; EDE-Q, Eating Disorder Examination Questionnaire.

Participants were screened for depression and eating disorders, using self-report questionnaires at baseline and week 4, 16, and 52 (Figure 1). Participants were monitored for disordered eating by trained study dietitians during each consultation according to a prespecified protocol (Figure 1).^26^ If support for depression or disordered eating was needed, this was provided as part of the study or referral to external services as per standard clinical care. Additional support visits for disordered eating were recorded. All adolescents were referred to their primary care practitioner for ongoing care at week 52.

### Measures

The Center for Epidemiologic Studies Depression Scale–Revised 10-Item Version for Adolescents (CESDR-10; scores 0-30),^29^ Eating Disorder Examination Questionnaire (EDE-Q; scores 0-6),^31^ and Binge-Eating Scale (BES; scores 0-46)^32^ were used. CESDR-10 scores of 8 or greater indicate subthreshold symptoms of depression, with a possible, probable, and major depressive episode, defined by the presence of anhedonia, dysphoria, or irritability with additional symptoms (eTable 1 in Supplement 4).^30^ An EDE-Q cutpoint of 2.7 ^33^ or the presence of disordered eating behaviors was used to identify adolescents at risk of eating disorders (Figure 1). On the BES, a score of 17 or less indicates no binge eating, scores 18 to 26 indicate mild/moderate binge eating, and scores of 27 or greater indicate severe binge eating.^32^ The eMethods in Supplement 4 contains additional details.^33^

### Statistical Analysis

Statistical analyses were performed using SPSS statistics, version 28.0 (IBM Corp) blinded to group allocation. All participant data were retained, consistent with intention to treat for longitudinal analysis. Linear mixed models, with an autoregressive first-order covariance structure and restricted maximum likelihood, were used to estimate the change in outcomes between baseline and week 4, 16, and 52. Assumptions of modeling were met. Analyses were repeated using an unstructured covariance model as part of sensitivity analyses. A pairwise post hoc comparison was conducted to compare means over time. Results are presented as the difference in estimated marginal means (EMM). Nine sibling pairs were recruited. Analyses were repeated removing 1 participant from each pair, with no differences in results; therefore, all data were retained in analyses. Independent-sample *t* tests and Mann-Whitney *U* tests were used to test for differences between continuous variables at baseline, week 4, and week 16 between those who completed and those who did not complete the study. For categorical variables, descriptive statistics were used to describe the number of participants meeting screening criteria at each time point and those above and below prespecified cutpoints at baseline and week 52. These proportions are presented for those with complete data at each time point. The content of additional support visits provided to adolescents was independently coded by 2 authors, with agreement reached through discussion.

## Results

In total, 141 adolescents (median [range] age, 14.8 [12.9-17.9] years; 70 female [49.6%]; 71 male [50.4%]) enrolled, and 97 (48 female [49.5%]) completed the 52-week intervention (eFigure 1 in Supplement 4). A total of 117 of the 141 participants (82.9%) were born in Australia, 17 (12.1%) were born in another country, 67 (47.5%) reported 1 or both parents were born outside of Australia, 57 (40.4%) reported speaking a language other than English at home, and 2 (1.4%) identified as Aboriginal or Torres Strait Islander. Seven participants declined to respond. Adolescents had a mean (SD) BMI of 35.39 (4.17; calculated as weight in kilograms divided by height in meters squared) and a BMI *z* score of 2.40 (0.46) at baseline.^27^ At baseline, adolescents had median scores of 9.00 (IQR, 4.00-14.50) on CESDR-10, 2.28 (IQR, 1.43-3.14) on EDE-Q, and 11.00 (IQR, 5.00-17.00) on BES, with no difference between groups.

### Depression

#### Change Over Time

**Figure 2.**
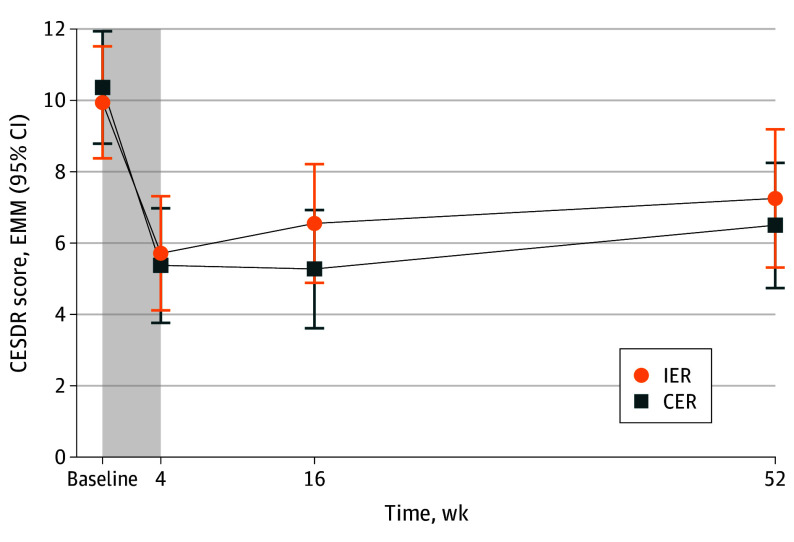
Change in Symptoms of Depression Between Baseline and Week 52 Total number of participants analyzed was 141. Values are estimated marginal means (95% CI). Shaded area represents the 4-week very low energy diet phase of the trial. CER indicates continuous energy restriction; CESDR-10, Center for Epidemiologic Studies Depression Scale–Revised 10-Item Version for Adolescents; EMM, estimated marginal means; IER, intermittent energy restriction.

There was an initial reduction in CESDR-10 scores at week 4 (change in EMM IER, −4.23; 95% CI, −5.66 to −2.81; change in EMM CER, −4.99; 95% CI, −6.42 to −3.57) and maintained at week 52 (IER, −2.70; 95% CI, −4.95 to −0.45; CER, −3.87; 95% CI, −5.98 to −1.77) with no difference between groups (mean difference at week 52, 0.75; 95% CI, −1.86 to 3.37) (Table and Figure 2). There were no differences between those who completed and those who did not complete the study at any time point.

**Table.** Mean Risk Scores for Self-Report Symptoms of Depression, Eating Disorder Risk, and Binge Eating and Difference Between Intervention Groups^a^

Variable; time	Estimated marginal mean (95% CI)		Difference in means between groups (95% CI)
	IER	CER	
**CESDR-10, EMM (95% CI)** ^b^			
Baseline	9.95 (8.39 to 11.51)	10.37 (8.80 to 11.95)	−0.42 (−2.64 to 1.80)
4 wk	5.72 (4.11 to 7.32)	5.38 (3.77 to 6.99)	0.34 (−1.93 to 2.61)
16 wk	6.56 (4.89 to 8.22)	5.28 (3.62 to 6.93)	1.28 (−1.07 to 3.63)
52 wk	7.25 (5.32 to 9.19)	6.50 (4.74 to 8.26)	0.75 (−1.86 to 3.37)
**Total EDE-Q, EMM (95% CI)** ^c^			
Baseline	2.40 (2.14 to 2.66)	2.31 (2.05 to 2.57)	0.09 (−0.28 to 0.46)
4 wk	1.73 (1.47 to 2.00)	1.85 (1.58 to 2.12)	−0.12 (−0.49 to 0.26)
16 wk	1.76 (1.49 to 2.04)	1.66 (1.38 to 1.93)	0.10 (−0.29 to 0.49)
52 wk	1.77 (1.46 to 2.09)	1.75 (1.46 to 2.04)	0.02 (−0.41 to 0.45)
**Shape concern, EMM (95% CI)**			
Baseline	3.34 (2.96 to 3.71)	3.24 (2.87 to 3.62)	0.09 (−0.43 to 0.62)
4 wk	2.05 (1.67 to 2.43)	2.14 (1.76 to 2.52)	−0.09 (−0.63 to 0.45)
16 wk	2.09 (1.70 to 2.48)	1.82 (1.43 to 2.21)	0.27 (−0.28 to 0.83)
52 wk	2.28 (1.83 to 2.73)	2.06 (1.65 to 2.48)	0.22 (−0.40 to 0.83)
**Weight concern, EMM (95% CI)**			
Baseline	3.16 (2.84 to 3.48)	3.08 (2.76 to 3.40)	0.08 (−0.37 to 0.53)
4 wk	1.98 (1.65 to 2.30)	2.01 (1.69 to 2.34)	−0.04 (−0.50 to 0.42)
16 wk	2.11 (1.78 to 2.45)	1.86 (1.53 to 2.19)	0.25 (−0.22 to 0.73)
52 wk	2.21 (1.83 to 2.60)	2.16 (1.80 to 2.51)	0.06 (−0.47 to 0.58)
**Eating concern, EMM (95% CI)**			
Baseline	1.62 (1.36 to 1.88)	1.48 (1.21 to 1.74)	0.14 (−0.23 to 0.52)
4 wk	0.72 (0.45 to 0.99)	0.76 (0.48 to 1.03)	−0.04 (−0.42 to 0.35)
16 wk	0.97 (0.69 to 1.26)	0.84 (0.56 to 1.19)	0.14 (−0.26 to 0.54)
52 wk	1.09 (0.75 to 1.42)	1.07 (0.77 to 1.37)	0.02 (−0.43 to 0.47)
**Dietary restraint, EMM (95% CI)**			
Baseline	1.48 (1.15 to 1.80)	1.43 (1.10 to 1.75)	0.05 (−0.41 to 0.51)
4 wk	2.18 (1.84 to 2.51)	2.48 (2.15 to 2.82)	−0.31 (−0.78 to 0.17)
16 wk	1.89 (1.54 to 2.24)	2.12 (1.78 to 2.47)	−0.23 (−0.72 to 0.26)
52 wk	1.57 (1.15 to 1.99)	1.70 (1.33 to 2.08)	−0.13 (−0.69 to 0.42)
**BES score, EMM (95% CI)** ^d^			
Baseline	11.85 (10.04 to 13.67)	11.42 (9.59 to 13.67)	0.43 (−2.14 to 3.01)
4 wk	7.47 (5.62 to 9.32)	7.98 (6.12 to 9.83)	−0.51 (−3.13 to 2.11)
16 wk	8.08 (6.18 to 9.98)	7.20 (5.31 to 9.10)	0.88 (−1.81 to 3.56)
52 wk	8.13 (5.96 to 10.31)	11.04 (9.04 to 13.05)	−2.91 (−5.87 to 0.05)

Abbreviations: BES, Binge Eating Scale; CER, continuous energy restriction; CESDR-10, Center for Epidemiological Studies Depression Scale–Revised 10-Item Version for Adolescents; EDE-Q, Eating Disorder Examination Questionnaire; IER, intermittent energy restriction.

^a^ Intention-to-treat analysis using linear mixed models. Values are estimated marginal means (95% CI).

^b^ CESDR-10 score ranged from 0 to 30. Total CESDR-10 participant number was 141.

^c^ EDE-Q score ranged from 0 to 6 for global scores and subscales. Total EDE-Q participant number was 141.

^d^ BES score ranged 0 to 46 (0-17 no binge eating; 18-26 mild/moderate binge eating; ≥27 severe binge eating). Total BES participant number was 139.

#### Symptom Classification

At baseline, 31 of 141 adolescents (22%) were classified as having a possible, probable, or major depressive episode in the 7 days before enrollment (eTable 2 and eFigure 2 in Supplement 4), with 47 (33%) reporting subthreshold symptoms. At week 52, the proportion of adolescents who completed the study classified as having a possible, probable, or major depressive episode was 8 of 92 (9%), with 60 (65%) reporting no symptoms. Five adolescents (5%; 1 IER, 4 CER) who reported no symptoms of depression at baseline had an increase in scores. Four adolescents reported subthreshold symptoms of depression, and 1 adolescent, with long-standing depression, met criteria for a probable depressive episode in the past 7 days.

### Eating Disorders

#### Change Over Time

**Figure 3.**
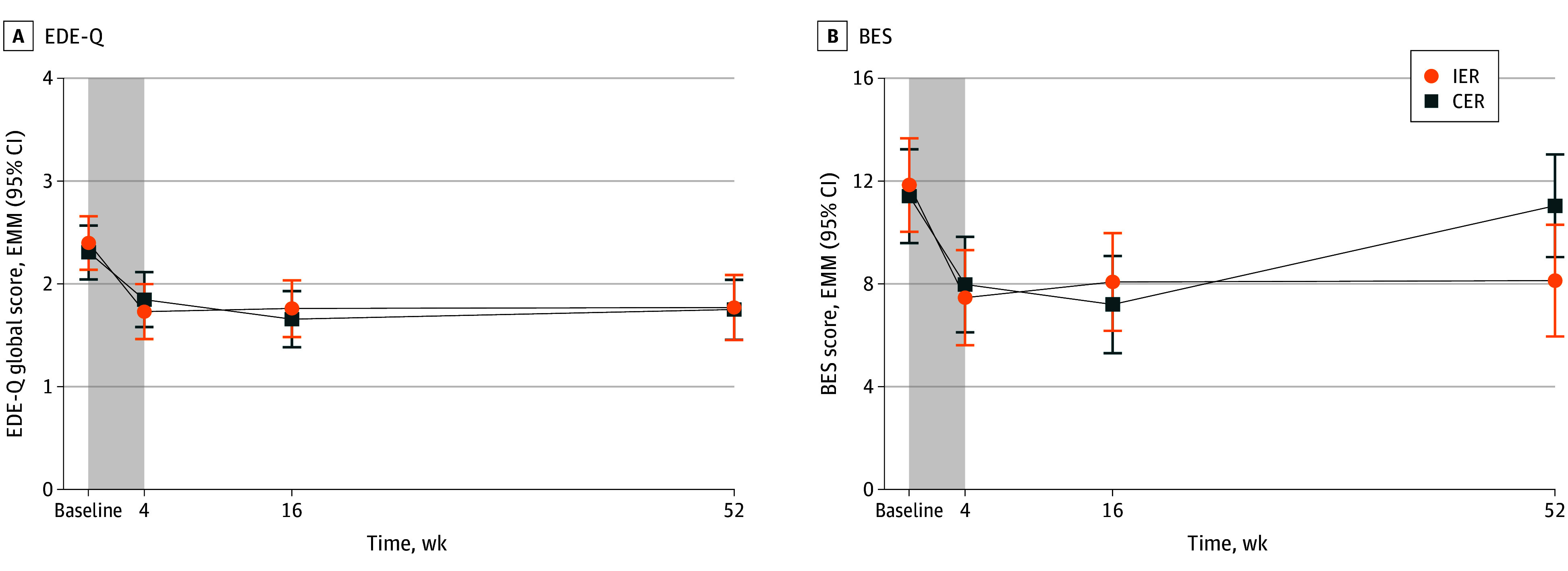
Change in Symptoms of Eating Disorders and Binge Eating Between Baseline and Week 52 A, Eating disorders between baseline and week 52. B, Binge eating between baseline and week 52. Values are estimated marginal means (95% CI). Shaded area represents the 4-week very low energy diet phase of the trial. Total number of participants was 141 for the Eating Disorder Examination Questionnaire (EDE-Q) and 139 for the Binge Eating Scale (BES). CER indicates continuous energy restriction; EMM, estimated marginal means; IER, intermittent energy restriction.

There was an initial reduction in EDE-Q global score at week 4 (IER, −0.67; 95% CI, −0.89 to −4.44; CER, −0.46; 95% CI, −0.68 to −0.24) and maintained at week 52 (IER, −0.63; 95% CI, −0.97 to −0.26; CER, −0.56; 95% CI, −0.89 to −0.22) with no difference between groups (mean difference at week 52, 0.02; 95% CI, −0.41 to 0.45) (Table and Figure 3A). There were no differences between those who completed and those who did not complete the study at any time point.

#### Subscale Scores

**Figure 4.**
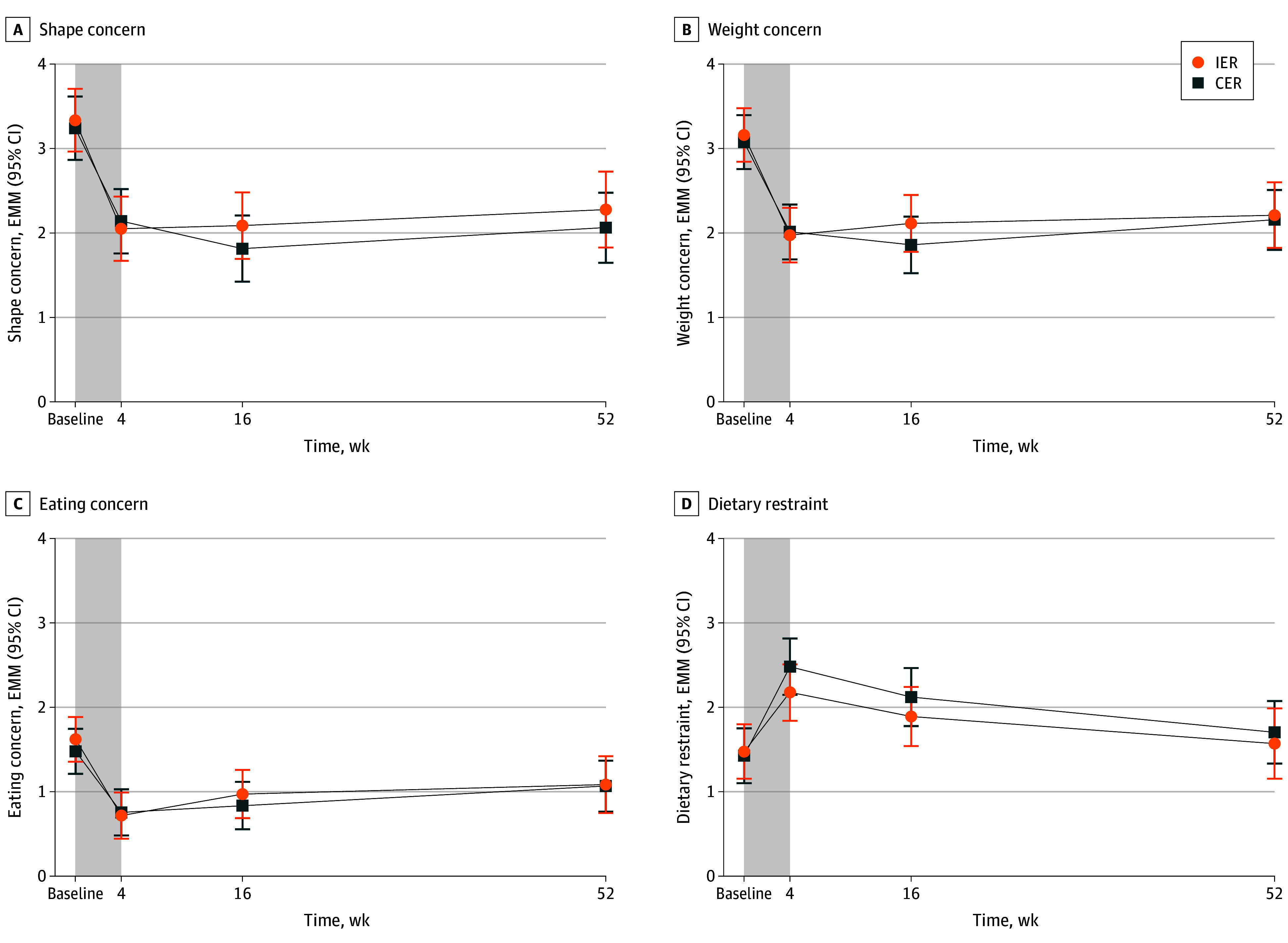
Change in Shape Concern, Weight Concern, Eating Concerns, and Dietary Restraint Subscales of the Eating Disorder Examination Questionnaire (EDE-Q) Between Baseline and Week 52 A, Shape concern. B, Weight concern. C, Eating concern. D, Dietary restraint. Values are estimated marginal means (95% CI). Shaded area represents the 4-week very low energy diet phase of the trial (total participants, n = 141). CER indicates continuous energy restriction; EMM, estimated marginal means; IER, intermittent energy restriction.

At week 4, there was an initial reduction in subscale scores for shape (IER, −1.28; 95% CI, −1.59 to −0.98; CER, −1.10; 95% CI, −1.41 to −0.79), weight (IER, −1.19; 95% CI, −1.46 to −0.91; CER, −1.06; 95% CI, −1.34 to −0.79), and eating concern (IER, −0.90; 95% CI, −1.17 to −0.63; CER, −0.72; 95% CI, −1.00 to −0.45), which were maintained at week 52, with no difference between groups (Table and Figures 4A-C). Dietary restraint increased at week 4 (IER, 0.70; 95% CI, 0.34-1.06; CER, 1.05; 95% CI, 0.69-1.42]), returning to baseline by week 52 with no difference between groups (Table and Figure 3D).

#### Disordered Eating Symptoms

At baseline, 110 of 141 adolescents (78%) reported any symptom of disordered eating as defined in Figure 1. Overeating (94 of 141 [67%]), binge eating (70 of 141 [50%]), and loss of control (58 of 141 [41%]) were most frequently reported (eTable 2 and eFigure 3 in Supplement 4). At week 52, the proportion of participants who completed the study and reported any symptom of disordered eating was 56 of 92 (61%).

#### EDE-Q Cutpoint

For those who completed the study and using an EDE-Q cutpoint of 2.7, a total of 51 adolescents (55%) remained below the cutpoint for the duration of the trial, and 8 (9%) remained above the cutpoint. There were 25 adolescents (27%) who had scores of 2.7 or greater at baseline but did not at week 52. A total of 8 adolescents (9%; 3 IER, 5 CER) had an increase in scores from less than 2.7 at baseline to 2.7 or greater at week 52. Of the 8 adolescents who had increased scores, 4 had psychological comorbidity at baseline and were already engaged with, or referred to, external psychological support. The remaining 4 adolescents were not identified as having overt concerns with disordered eating, although strategies to manage overeating were discussed.

### Binge Eating

#### Change Over Time

At 4 weeks, there was a reduction in BES scores (IER, −4.38; 95% CI, −5.93 to −2.83; CER, −3.44; 95% CI, −5.00 to −1.88), maintained in IER at 52 weeks (−3.72; 95% CI, −6.20 to −1.24) but not CER (−0.38; 95% CI, −2.71 to 1.96), with no difference between groups (mean difference at week 52, −2.91; 95% CI, −5.87 to 0.05) (Table and Figure 3B). There were no differences between those who completed and those who did not complete the study at any time point.

#### Binge-Eating Classification

At baseline, 28 adolescents (21%) were classified as having mild/moderate or severe binge eating (eTable 2 and eFigure 4 in Supplement 4). At week 52, 15 of 92 adolescents (16%) who completed the study reported mild/moderate or severe binge eating. Sixty-five adolescents (71%) reported no binge eating at baseline and week 52. Four adolescents (4%) reported mild/moderate binge eating at both time points. Ten adolescents (11%) with mild/moderate binge eating and 2 (2%) with severe binge eating at baseline reported no binge eating at week 52. Ten adolescents (11%) experienced an increase in binge eating, with 8 (4 in each group) categorized as not binge eating at baseline moved to mild/moderate (n = 6) or severe (n = 2) binge eating at week 52. Two adolescents (CER group) increased from mild/moderate to severe binge eating. Of the 10 adolescents with an increase in binge eating, 5 had other mental health comorbidities identified at baseline or during the trial (eg, autism spectrum disorder, depression, anxiety, mild uncontrolled eating) and were referred for external psychological support or provided with additional support during the trial. On further assessment, 5 adolescents were not identified as having overt binge-eating symptoms; overeating behaviors were discussed with 3 adolescents.

### Outcome of Screening and Monitoring

Seventeen adolescents (12.1%) required support or referral for depression and/or disordered eating.

#### Screening Questionnaires

At baseline, 7 adolescents (5%) were identified as requiring support for disordered eating and 4 (2.8%) for depression. This was provided during the trial if they were not already engaged with external support. At week 52, 2 of the adolescents who were identified at baseline and 1 additional adolescent required referral for ongoing psychological support for disordered eating.

#### Dietetic Monitoring

Seven of 141 adolescents (5%, all female; 5 IER; 2 CER) were identified via dietetic monitoring as requiring additional support for disordered eating or body image concerns. They received a median of 3 (range, 1-9) additional sessions with the dietitian, clinical psychologist, and/or pediatrician, often with multidisciplinary input. Additional support was mostly provided during phase 3 of the study, when scheduled reviews reduced. Of those requiring additional support, 1 adolescent had mild body image concerns after a sports injury, with no disordered eating concerns. Two adolescents had support for meal skipping with mild body image concerns or secret eating, together with excess weighing, both in the context of requiring psychological support from baseline for anxiety and social difficulties, respectively. One adolescent had additional support to address body dissatisfaction and rigidity around diet and exercise. Three adolescents received additional support for restriction or rigidity around eating, with rapid weight loss; 2 of these adolescents had a medical review for amenorrhea, which resolved during the trial.

### Adverse Events

Two adolescents had adverse events related to mental health and were withdrawn. One adolescent was withdrawn at week 14 after the reemergence of prior body image concerns and related poor self-esteem. The second adolescent was diagnosed with atypical anorexia nervosa associated with excess restriction and rapid weight loss (identified via monitoring protocol, as reported previously), despite transition to weight maintenance plan, and was withdrawn at week 22 as a severe adverse event. This occurred during the COVID-19 pandemic when review appointments were predominantly conducted virtually. Both adolescents received ongoing mental health care.

## Discussion

The aim of these analyses was to evaluate changes in self-reported symptoms of depression, eating disorders, and binge eating during the Fast Track to Health trial. At baseline, over one-half of adolescents reported some symptoms of depression and/or eating disorders, with 21% reporting mild, moderate, or severe binge eating. Mean scores on the CESDR-10, EDE-Q, and BES scales were reduced at week 4, after the VLED phase, and maintained at week 52 for depression and eating disorder risk, with no difference between groups. The reduction in BES score at week 4 was only maintained in the IER group at 52 weeks. A proportion of adolescents (12.1%) required additional support for depression or disordered eating, identified through self-report questionnaires or dietetic monitoring, with support for appropriate early intervention. One adolescent developed an eating disorder, occurring during the COVID-19 lockdown restrictions, making it difficult to isolate the influence of the intervention. Overall, participation in intensive behavioral interventions using a prescriptive dietary approach did not worsen mental health or eating behaviors. Indeed, an improvement in self-report symptoms was seen alongside relative reductions in BMI, and improved blood pressure percentile, total cholesterol level, and triglyceride level in both groups.^27^ This is particularly important as the trial was predominantly undertaken during the COVID-19 pandemic, with intermittent lockdowns and other public health restrictions, which also impacted adolescent depression, disordered eating, and BMI.^6^

Symptoms of depression and disordered eating reduced, and did not increase, during the intervention, which is consistent with prior reviews^16^ and trials of alternate-day fasting and time-limited eating in adults and adolescents.^39^ Interestingly, the greatest reduction in scores occurred after the VLED phase, with no further reduction after transition to CER and IER. These outcomes have scarcely been measured during adolescent VLED interventions.^42^ Findings are consistent with adult VLED trials that show reduced binge eating,^43^ but are in contrast to longitudinal studies in adolescents showing higher degrees of energy restriction, are associated with disordered eating.^23^ Given the short 4-week VLED phase, it is difficult to speculate why this initial improvement was seen. It is possible that the initial reduction in BMI,^27^ together with frequent support,^11^ improved mental well-being. However, eating disorders can take several years to emerge.^44^ Further research is needed on mental health effects of VLED interventions and maintenance beyond 1 year.

Although adolescence is a time of increased prevalence of depression and disordered eating,^3^ and symptoms increased during COVID-19 pandemic,^6^ few adolescents had increased symptoms during the trial. Those who experienced elevated symptoms often had other mental health concerns at enrollment. To our knowledge, few studies have reported the onset of eating disorders during adolescent obesity treatment. One trial^45^ reported 2 cases of eating disorders from 125 adolescents receiving liraglutide, 3.0 mg, with lifestyle therapy, and another^46^ reported 3 cases of binge eating disorder at 6-year follow-up of a behavioral program. More data are needed to understand the proportion of adolescents who may be at risk of eating disorders while receiving obesity treatment.^47^ Addressing participants’ mental health within obesity programs, or enabling concurrent referral for psychological support, is important to support holistic health of adolescents during weight management.^1^

Prescriptive dietary interventions, such as those used in this trial, are considered dieting behaviors that may precede eating disorder development.^12^ Dietary restraint scores increased during the intervention period, as might be expected due to the energy restriction targets, and returned to baseline at 52 weeks. Given that shape concern, weight concern, and depression all reduced during this time, the transient increase in dietary restraint is unlikely to represent ongoing problematic eating.^21^ Additionally, mean dietary restraint scores remained well below those of clinical samples of adolescents (4.00)^51^ or adults (4.17)^52^ with anorexia nervosa, even after the VLED phase of the intervention (2.18, IER group; 2.48, CER group) (Table). At 52 weeks, mean dietary restraint scores had returned to baseline and were similar to community norms for adolescent girls.^53^ This suggests that dietary prescription in the context of a multidisciplinary care poses less risk than when undertaken without supervision in the community. We speculate that this is due to the multidisciplinary nature of the intervention and frequency of support provided by trained clinicians. However, further research is needed to directly compare different dietary interventions, no-treatment controls, and community samples to understand the association between different diets and eating disorder risk.

Use of self-report questionnaires to screen for depression and eating disorders, particularly at baseline, enabled adolescents requiring support to receive this early. Clinical monitoring for emergence of disordered eating during dietetic reviews enabled a subgroup of adolescents with potentially problematic behaviors to be identified and assessed. Of note, some symptoms identified during dietetic monitoring, including rigidity and obsessionality or fixation with numerical changes, eg, weight and calories, are not included in the EDE-Q, with different adolescents identified by each approach. This highlights the need for regular contact with clinicians who can verbally assess behaviors. Screening and monitoring for depression and eating disorders as part of obesity treatment facilitates opportunistic identification of a high-risk group that infrequently accesses health services.^55^ Identification of adolescents at risk may enable clinicians to facilitate access to mental health services, resulting in the provision of additional support. Initiating treatment of potential triggers or contributors to obesity may also support weight management attempts or improve treatment outcomes. It is important to ensure that clinicians can interpret self-report questionnaires and use them to guide clinical decision making. Additionally, the time, resources, and availability of services are important considerations in evaluating feasibility of conducting universal screening and monitoring in a clinical setting.^56^

### Strengths and Limitations

Our study has several strengths. This was the first, to our knowledge, in-depth study of depression and eating-disorder symptoms in treatment-seeking, culturally diverse, Australian adolescents with obesity and concurrent complications. Structured screening and monitoring procedures were used with evidence-based, predefined cutpoints. 

This study also has limitations. Data presented are secondary analyses using self-report measures, therefore, results should be interpreted with caution. Two of the 4 subscales within the EDE-Q relate to body dissatisfaction, which is elevated in adolescents with obesity, and a third includes dietary restriction, which is a component of the intervention.^12^ Adolescents also reported misunderstanding questions on the EDE-Q. For example, participants reported occasions of overeating based on consuming a somewhat larger portion size at main meals compared with other family members or peers. Questions related to vomiting were sometimes misunderstood, eg, vomiting due to migraines or gastroesophageal reflux were reported but were not self-induced vomiting. Although the EDE-Q is validated for use in adolescents, a youth-specific version of this questionnaire^35^ or more recently validated short eating-disorder assessments for youth,^57^ may improve screening in future trials. The trial was mainly undertaken during the COVID-19 pandemic. Therefore, an increase in self-reported depression and disordered eating during this time could be due to the intervention or the COVID-19 pandemic and differentiation between these is not possible.

## Conclusions

In this randomized clinical trial, adolescents with obesity who were seeking treatment self-reported symptoms of depression, eating disorders, and/or binge eating. Symptoms reduced for most adolescents, concurrent with BMI reduction, highlighting a potential dual role of obesity treatment in improving mental as well as physical health. A subgroup of adolescents were identified as needing additional support for depression or disordered eating with some experiencing increased symptoms during the intervention. Approaches to screening and monitoring using a combination of questionnaires and clinician assessment appear important. Treatment practitioners should have mechanisms in place for identification and management of changes in psychopathology in adolescents seeking obesity treatment. Although obesity services may present an opportune time to support adolescent mental health, appropriate training, time, and resourcing is needing to implement this into practice.

## Notes

Supplement 1.
Trial Protocol.

Supplement 2.
Trial Protocol Amendments.

Supplement 3.
Statistical Analysis Plan.

Supplement 4. **eMethods.** Screening **eTable 1.** The Center for Epidemiologic Studies Depression Scale–Revised 10-Item Version for Adolescents Cut Points Based on the Scoring Described by Haroz et al **eFigure 1.** CONSORT Figure **eTable 2.** Proportion of Adolescents With Symptoms of Depression, Eating Disorders, and Binge Eating and Change Over Time by Group **eFigure 2.** Proportion of Adolescents Meeting Depression Screening Criteria Over Time, All Participants Combined **eFigure 3.** Proportion of Adolescents Meeting Eating Disorder Screening Criteria Over Time, All Participants Combined **eFigure 4.** Proportion of Adolescents Meeting Binge Eating Scale Criteria Over Time, All Participants Combined
eReferences

Supplement 5.
Data Sharing Statement.
