# Supplementary material for: Symptoms of Depression, Eating Disorders, and Binge Eating in Adolescents With Obesity: The Fast Track to Health Randomized Clinical Trial
Source: JAMA Pediatr. 2024 Aug 26:e242851. Online ahead of print. doi: 10.1001/jamapediatrics.2024.2851 (PMC11348093; doi:10.1001/jamapediatrics.2024.2851)
Supplement: Supplement 1. — Trial Protocol. [file jamapediatr-e242851-s001.pdf]

Fast Track to Health
The intermittent energy restricted diet in adolescents with obesity: a randomised controlled trial

PROTOCOL

Version: 3.3
Date: 02/12/2022

Authors

Professor Louise Baur; Associate Professor Sarah Garnett; Professor Christopher Cowell; Dr Natalie Lister; Dr Megan Gow; Dr Shirley Alexander; Professor Helen Truby; Professor Clare Collins; Professor Susan Paxton; Ms Hiba Jebeile

Investigators

Professor Louise Baur; Professor Helen Truby; Associate Professor Sarah Garnett; Associate Professor Krista Varady; Professor Christopher Cowell; Professor Clare Collins; Professor Susan Paxton; Dr Natalie Lister; Dr Megan Gow; Dr Justin Brown; Dr Shirley Alexander; Ms Kerryn Chisholm; Ms Alicia Grunseit; Ms Hiba Jebeile

Sponsor/s

National Health and Medical Research Council - Project grant APP1128317

CONFIDENTIAL

This document is confidential and the property of University of Sydney. No part of it may be transmitted, reproduced, published, or used without prior written authorisation from the institution.

Statement of Compliance

This document is a protocol for a research project. This study will be conducted in compliance with all stipulation of this protocol, the conditions of the ethics committee approval, the NHMRC National

Statement on ethical Conduct in Human Research (2007) and the Note for Guidance on Good Clinical Practice (CPMP/ICH-135/95).

Table of Contents

1 Glossary of Abbreviations & Terms....4

2 Study Investigators....4

3 Study Sites....5

3.1 Study Location/s....5

4 Funding and Resources....6

4.1 Source/s of Funding....6

5 Introduction/Background Information....6

5.1 Lay Summary....6

5.2 Introduction....7

5.3 Background information....7

6 Study Objectives....9

6.1 Primary Objectives....9

6.2 Secondary Objectives....9

6.3 Outcome Measures....10

6.3.1 Primary Outcome....10

6.3.2 Secondary Outcome....11

7 Study Design....13

7.1 Study Design Diagram....13

7.2 Study Type & Design & Schedule....14

7.2.1 Study Design....14

7.2.2 Study Description....14

7.2.3 Intervention Fidelity....19

7.2.4 Fitbit®....23

7.2.5 Expected duration of the study 24
7.2.6 Contingency plans 31
7.2.7 Student involvement 31
7.3 Standard Care and Additional to Standard Care Procedures 31
7.4 Randomisation 32
7.5 Study methodology 32
7.6 Recruitment Procedure 37
7.6.1 Recruitment 37
7.6.2 Retention strategies 38
7.7 Inclusion Criteria 38
7.8 Exclusion Criteria 38
7.9 Screening Assessment 39
7.10 Consent 40
8 Participant Safety and Withdrawal 40
8.1 Risk Management and Safety 40
8.2 Adverse Event Reporting 40
8.2.1 Definitions 40
8.2.2 Assessment and Documentation of Adverse Events 40
8.2.3 Eliciting Adverse Event Information 41
8.3 Handling of Withdrawals 41
8.4 Replacements 42
9 Statistical Methods 42
9.1 Sample Size Estimation & Justification 42
9.2 Power Calculations 42
9.3 Statistical Methods to Be Undertaken 42
10 Storage of Blood and Tissue Samples 43
10.1 Details of where samples will be stored, and the type of consent for future use of samples 43
11 Data Security & Handling 43

11.1 Details of where records will be kept & how long will they be stored 43

11.2 Confidentiality and Security 44

11.3 Ancillary data 44

12 Appendix 45

13 References 48

1 Glossary of Abbreviations & Terms

Abbreviation	Description (using lay language)
BIA	Bioelectrical impedance analysis. A body composition technique to assess total body water from which body fat can be derived.
IER	Intermittent energy restriction. It involves three "energy restricted" days each week, consuming one-third of daily energy requirements (~2520–2940 kJ or 600–700 kcal), and consuming a healthy diet for the four "feeding" or "regular eating" days.
VLED	Very low energy diet. Participants choose to either consume four Optifast® meal replacements per day (shakes and/or soups, and/or bars, and/or desserts) or three Optifast® meal replacements and one meal consisting of 100–150 g lean, cooked meat and carbohydrate-free vegetables. The diet contains ~800 kcal/day.

2 Study Investigators

Investigator Position	Name	Institution
Principal Investigator	Professor Louise Baur	The Children's Hospital at Westmead, Westmead, Australia<br>University of Sydney, Sydney, Australia
Chief Investigators	Professor Helen Truby	Monash University, Melbourne, Australia
	Associate Professor Sarah Garnett	The Children's Hospital at Westmead, Westmead, Australia<br>University of Sydney, Sydney, Australia

	Associate Professor Krista Varady	University of Illinois, Chicago, USA
	Professor Christopher Cowell	The Children's Hospital at Westmead, Westmead, Australia<br>University of Sydney, Sydney, Australia
	Professor Clare Collins	University of Newcastle, Newcastle, Australia
	Professor Susan Paxton	La Trobe University, Melbourne, Australia
	Dr Natalie Lister	The Children's Hospital at Westmead, Westmead, Australia,<br>University of Sydney, Sydney, Australia
	Dr Megan Gow	The Children's Hospital at Westmead, Westmead, Australia<br>University of Sydney, Sydney, Australia
	Dr Justin Brown	Monash Children's Hospital, Melbourne, Australia
Associate Investigators	Dr Shirley Alexander	The Children's Hospital at Westmead, Westmead, Australia
	Ms Kerryn Chisholm	The Children's Hospital at Westmead, Westmead, Australia
	Ms Alicia Grunseit	The Children's Hospital at Westmead, Westmead, Australia
	Ms Hiba Jebeile	The Children's Hospital at Westmead, Westmead, Australia<br>University of Sydney

3 Study Sites

3.1 Study Location/s

Site	Address	Contact Person	Contact details

The Children's Hospital at Westmead, Westmead, Australia	Weight Management Services -Outpatient Clinic, Corner of Hawkesbury Road and Hainsworth Street, Westmead NSW 2145, Australia	Professor Louise Baur	P: 02 9845 1903<br>E: louise.baur@health.nsw.gov.au
Monash University, Melbourne, Australia	Be Active Sleep Eat (BASE) Facility, Level 1, 264 Ferntree Gully Road, Notting Hill VIC 3168, Australia	Professor Helen Truby	P: 03 9902 4261<br>E: helen.truby@monash.edu
Monash Children's Hospital, Melbourne, Australia	The Paediatric Endocrine Nutrition Clinic, 246 Clayton Road, Clayton VIC 3168, Australia	Dr Justin Brown	P: 03 9594 2464<br>E: justin.brown@monashhealth.org

4 Funding and Resources

4.1 Source/s of Funding

Funding body	Funding Scheme	Title	Application number	Dates funded
National Health and Medical Research Council (NHMRC)	Project Grant	The alternate day fasting diet in adolescents with obesity: a randomised controlled trial	APP1128317	01/01/2017-31/12/2020
Sydney Medical School Medical Foundation	Part salary for Dr Lister	Novel dietary interventions and cardio-metabolic risk in adolescents with obesity		2017-2018

5 Introduction/Background Information

5.1 Lay Summary

One in four Australian adolescents has overweight or obesity. While short-term weight loss is possible, keeping the weight off long term is difficult.

Intermittent energy restriction (IER) which has demonstrated success in adults, includes 3 days/week of energy restriction and 4 days of a healthy diet. IER may be more sustainable and lead to greater weight loss than daily energy restriction.

This study will test whether IER is effective, safe and acceptable to adolescents. The study will involve adolescents aged 13-17 years, who are affected by obesity; they will be randomly allocated to receive either the IER, or a standard weight control diet. Adolescents will be followed for 52 weeks with regular dietitian reviews, data collected will include weight, height, body composition, heart and diabetes risk measures from blood samples, and psychological well-being information.

5.2 Introduction

One in four Australian adolescents has overweight or obesity, with prevalence rates continuing to rise. Effective treatment of obesity in this age group is vital. Dietary interventions are generally effective in treating obesity in the short term, but the best way to achieve weight loss longer term in adolescents is unknown. Young people with obesity find it very difficult to adhere to the routine of daily calorie restriction - a core element of current weight management programs.

A new diet intervention that has demonstrated success in adults is intermittent energy restriction (IER), a variant of which is the diet popularised as the "5:2 Diet". There are many variations of IER in the literature, approaches typically involve 2-6 days per week of intense energy restriction (i.e. consuming 25-35% of daily energy needs)alternated with days of healthy eating or ad libitum intake

Despite the current research interest in IER in adults, there have been no studies of its use in adolescents. There are potential safety concerns as to whether IER promotes the development of disordered eating and whether it is nutritionally adequate. However, the avoidance of every day calorie restriction, the overall lower energy intake that IER provides, and it's flexibility in terms of youth lifestyles makes IER potentially very appealing, and hence sustainable to adolescents. This study is the first randomised controlled trial (RCT) of IER in adolescents.

This study is a multisite RCT weight-loss intervention comparing IER with a standard hypocaloric diet in adolescents aged 13-17 years with obesity.

The study *challenges existing clinical paradigms* by proposing that IER can be implemented as an alternative to standard continuous energy restricted diets for adolescents with obesity.

5.3 Background information

Increasing prevalence of obesity in adolescence, especially of severe obesity

One in four Australian adolescents has overweight or obesity. In contrast to the situation in primary school-aged children, *prevalence rates in this age group have continued to increase* in recent years. Rates of severe obesity in adolescence have more than doubled in the past two decades [1].

Obesity in adolescence is often complicated by psychosocial distress and associated with a range of other health problems, including insulin resistance, orthopaedic disorders, high blood pressure, fatty liver disease, dyslipidaemia and type 2 diabetes [2, 3]. If untreated, obesity in adolescence is highly likely to persist into adulthood, with many attendant complications.

Effective treatment of obesity is needed

Effective treatment, not just prevention, of obesity in adolescence is vital. Our published systematic reviews of adolescent obesity treatment concluded that lifestyle interventions, including diet, lead to mild-to-moderate weight loss [4, 5], and improvements in cardiometabolic outcomes [5]. The reviews also report weight loss regardless of the diets' macronutrient content, indicating that *the primary goal of diet interventions for weight loss in adolescents should be adherence to a reduction in total energy intake*[6]. However, the optimal way to achieve adherence to such a diet has not been identified.

Results from previous weight management studies in adolescents

Our group has undertaken the largest number of clinical trials of adolescent obesity management in Australasia, in both hospital and community settings. Our studies have shown that:

- Decreases in BMI and BMI z-score are associated with improvements in measures of self-esteem, health-related quality of life and a range of cardiometabolic risk factors[6, 7]. These persist for 2 years from baseline and >18 months from the end of intensive weight-management treatment.
- Adolescents seeking treatment for obesity have a strong preference for structured eating plans rather than unstructured advice [8].
- Adolescents given a prescriptive diet (under the supervision of health professionals) show improvements in eating behaviours at 6 months, including a reduction in emotional eating and eating in response to external cues, and experience no adverse effect on dietary restraint [9].
- Weight loss at 3 months from baseline is a strong predictor of adherence to, and participation in, the treatment program and better weight outcomes at 2 years [10].
- Additional mHealth support strategies (such as phone coaching, SMS messages or emails) are very acceptable to young people involved in weight loss trials [11, 12].
- Adolescents with obesity and newly diagnosed type 2 diabetes who are given a closely supervised very low energy diet (VLED) have significant weight loss of 3.9% (range, 0.6-7.8%) at 4 weeks and 5.0% (range, 2.8% gain to 8.8% loss) at 8 weeks, with marked improvements in diabetes control [13]. However, the diet is severe and may not suit all as a longer term form of therapy.
- Adherence to a calorie restricted diet, rather than the type of diet prescribed, is key to successful weight loss and improved metabolic factors at 12 and 24 months, regardless of macronutrient content [10, 14-16].

IER diet in adults

A *new diet intervention* that has shown improved body composition and cardiometabolic profiles in adult populations in studies, is a type of IER called alternate day fasting (ADF) [17]. ADF involves alternating between ad libitum 'feeding' days and 'fasting' days, which typically consist of a single meal containing about 25% of the daily energy needs. Another type of IER, referred to as modified alternate day fasting (MADF) involves 3-4 days per week of energy restriction (i.e. consuming 25-35% of the daily energy needs), with adherence to healthy eating guidelines on feeding days. This is the type of IER that we will focus on. IER theoretically improves dietary adherence due to individuals feeling less restricted by including 'days off' from energy restriction.

Why might IER work in adolescents?

Compared to other dietary interventions, IER achieves a lower average energy intake by greatly restricting intake on 3 days per week, which is not usually counterbalanced on the remaining days [18]. This should result in greater weight loss compared to traditional energy restriction. In addition, IER eating plans tend to fit in well with youth lifestyles, allowing adolescents to attend social activities on 'days off'. We propose that long term adherence to the diet in adolescents is therefore increased. The combination of lower energy intake, the avoidance of every day calorie restriction and the flexibility of IER should make it appealing and acceptable to adolescents with obesity. We will explore participants' and parents' views of acceptability in post-RCT questionnaires.

Potential concerns about IER in adolescents

Adolescence is a time of growth and increased nutrient requirements. Restricting intake can limit the opportunity to consume vital nutrients e.g. calcium, iron and zinc. We have expertise in dietary modelling using foods acceptable to this age group, and have developed IER meal plans to ensure nutritional adequacy.

Clinicians and parents are often concerned about restricting the diets of adolescents with obesity due to a perceived risk of inducing or exacerbating existing eating disorders [19]. However, our previous research suggests adolescents with obesity given a prescriptive diet under the supervision of health professionals, have normalised eating behaviours and improved quality of life [9]. We will explore the impact of IER on participants' eating behaviours, quality of life and body image.

Pilot study

No trials of IER in adolescents have been published, but since popularisation of variations of IER (e.g. the '5:2' diet) in at least 50 diet books, there is clinical demand for its use by adolescent patients at The Children's Hospital at Westmead (Dr Shirley Alexander and Ms Kerryn Chisholm, personal communication). After initial occasional clinical use, we initiated a 12 week pilot study of IER in 8 adolescents with obesity, using a pre-post study design. The mean weight change was 2.7kg, without the use of an initial VLED "jump-start" phase. Data from our clinic audits suggest that the diet is popular among adolescents with obesity, but it is not known if IER is superior to other best-practice dietary interventions in an adolescent population.

6 Study Objectives

6.1 Primary Objectives

To determine if IER results in lower body mass index (BMI) z-score after 52 weeks of intervention in adolescents with obesity compared with a standard, usual care, hypocaloric dietary intervention.

6.2 Secondary Objectives

Applicable at the Children's Hospital at Westmead, Monash Children's Hospital and Monash University

To determine if IER results in improvements in body composition; diet quality, food choices and food patterns; cardio-metabolic risk factors; physical activity; sleep and psycho-behavioural measures in adolescents with obesity compared with a standard, usual care, hypocaloric dietary intervention.

Applicable only at Monash Children's Hospital and Monash University

To determine if IER results in improvements in biomarkers of inflammation, and changes in gene expression and resting energy expenditure in adolescents with obesity, compared with a standard, usual care, hypocaloric dietary intervention.

This study is an opportunity to examine whether there are any differential impacts of the two diets on gene expression. The development of personalised nutrition is still in its infancy but the longer term perspective is that doctors may be able to predict who may respond better to specific dietary patterns [20]. This sub-study is a step towards this long term goal, made possible by advances in genomic sequencing. It takes the initial step by examining if and what genes are differentially expressed (changes in the epigenome) after weight loss per se and to establish whether there are differences between those that lose a clinically significant amount of weight and those who do not. The primary aim is to examine if there is any association between gene expression and weight loss in adolescents. This is an exploratory pilot study. This sub-study is part of Ms Kaitlin Day's PhD, supervised by Professor Melissa Southey, Chair of Precision Medicine at Monash Health Translational Research Precinct.

6.3 Outcome Measures

The assessment schedule is outlined in Table 1 and assessment methods in section 7.5 Study Methodology.

6.3.1 Primary Outcome

BMI z-score at 52 weeks.

6.3.2 Secondary Outcome

The following outcome measures performed at week 16 and 52, and at 24-months follow-up will be used to answer study questions

Outcome Measures	Applicable At:<br>The Children's Hospital At Westmead<br>Monash Children's Hospital<br>Monash University	Applicable Only At:<br>Monash Children's Hospital Monash University
**Body composition**	Weight<br>BMI as a % of the 95th centile for age<br>Waist-to-height ratio<br>Fat mass	
**Diet**	Diet quality<br>Food Choices<br>Food Patterns<br>Diet acceptability (Week 16 & 52 only)	
**Cardio-metabolic risk factors**	Systolic and diastolic blood pressure<br>Serum biochemistry (high-sensitivity CRP)<br>Lipid profile (total triglycerides, high-density and low-density cholesterol)<br>Glucose<br>Insulin<br>Liver function tests (LFTs)	
**Inflammatory markers**		(Week 16 & 52 only) Adipokines [Adiponectin, Leptin, Monocyte chemotactic protein-1 (MCP-1), Plasminogen activator inhibitor-1 (PAI-1) (Total) and Tumour necrosis factor-alpha (TNF-α)]<br>Cytokines [Interlleukin-1 beta (IL-1β), Interleukin-6 (IL-6) and Interleukin-8 (IL-8)]<br>Hepatocyte growth factor (HGF)<br>Nerve growth factor (NGF)<br>Resistin
**Psycho-behavioural**	Body dissatisfaction<br>Bulimic eating episodes<br>Psychological wellbeing<br>Weight related quality of life<br>Emotional wellbeing<br>External eating habits<br>Depression<br>Anxiety<br>Stress	

**Lifestyle**	Sedentary behaviour questionnaire<br>Physical activity questionnaire<br>Sleep quality questionnaire<br>Activity monitor (Fit Bit Flex 2)	
**Resting Energy Expenditure (Monash Only)**		Change of resting energy expenditure over time in relation to weight loss (performed at baseline, week 4 and week 16 and 52) and self-reported hunger and satiety (reported using visual analog scales for appetite and satiety at baseline, week 4, week 16 and week 52).
**Gene Expression**<br><br>**(Monash Only)**		To examine the association between gene expression and weight loss in adolescents. Gene expression will be measured by quantifying messenger RNA transcripts by RNA sequencing. (At baseline and week 16)

7 Study Design

7.1 Study Design Diagram

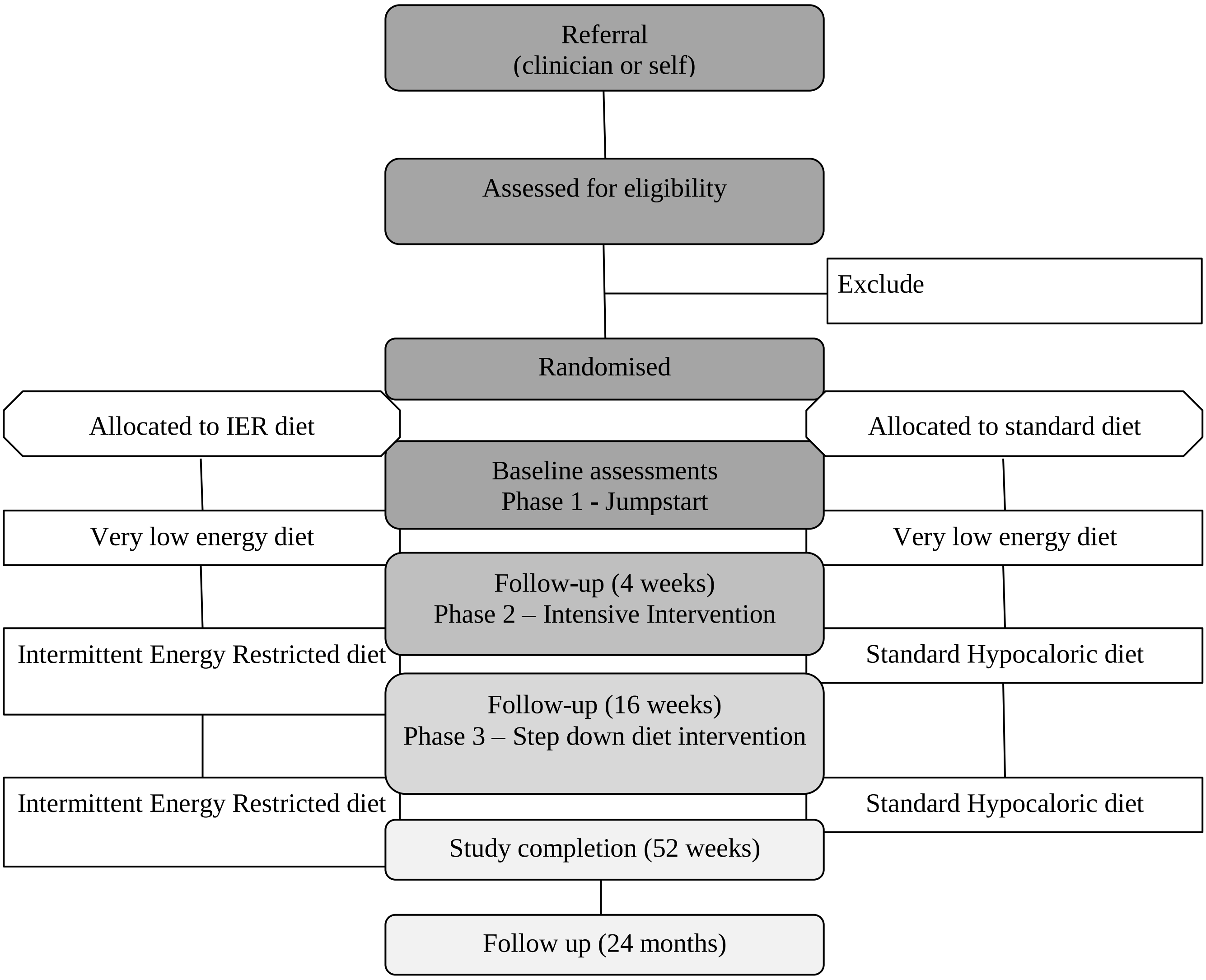

Figure 1

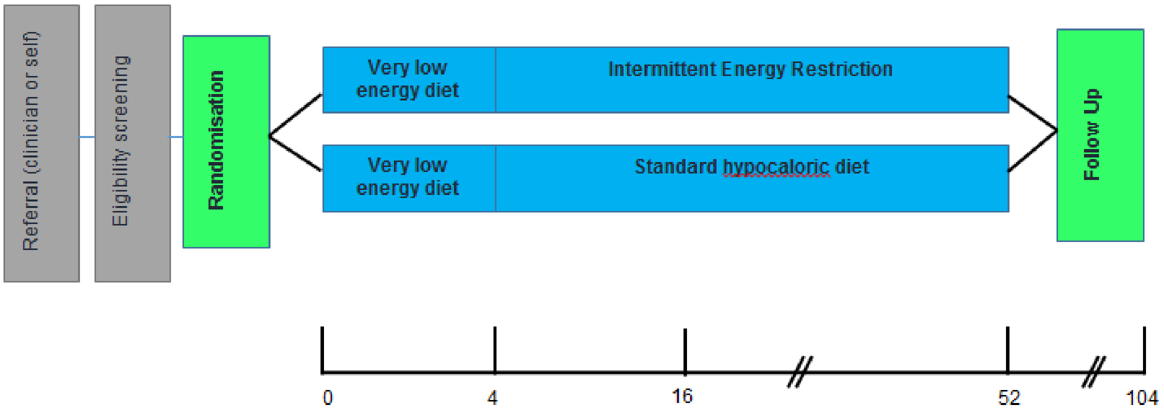

Figure 2

	Phase 1 Jumpstart	*Phase 2 Intensive Intervention*	*Phase 3 Step Down Diet Intervention*	*12-24 Month Follow Up*
Dietitian Consults (weeks)	*0, 1, 2, 3, 4*	*6, 9, 12, 16*	*20, 26, 36, 52*	*104*
Dietitian Support (weeks)	*1*	*8, 11, 14*	*18, 24, 28, 42, 48*	*58-60;78*

7.2 Study Type & Design & Schedule

7.2.1 Study Design

This is a prospective, randomised, multi-centre study designed to determine if IER results in lower BMI z-score after 52 weeks of intervention in adolescents with obesity, compared with a standard, usual care, hypocaloric dietary intervention. A total of 186 participants aged 13–17 years will be randomised on a 1:1 ratio to 4 weeks of VLED, followed by 48 weeks of IER (experimental arm), or 4 weeks of VLED, followed by 48 weeks of current best-practice calorie restricted diet (active control arm). To ensure even distribution, randomization will be stratified by clinical study site, age, sex and BMI. Randomisation will occur at baseline and participants will be informed of their allocated dietary patterns during phase 1.

7.2.2 Study Description

There are three phases in the intervention:

1. Jump-start weight loss
2. Intensive diet, and
3. Step-down diet

All participants will undergo an initial VLED jump-start phase (up to 4 weeks) and then transition to a standard dietary intervention (active control) or IER (experimental arm). The VLED jump-start phase achieves rapid, short-term weight loss, compared to commencing intensive diets immediately, and is frequently used in clinical practice. Short term weight loss predicts long term weight loss success[10], but VLEDs are severe diets and may not be sustainable long term.

Phase 1: Jump-start weight loss (Baseline, up to 4 weeks)

Participants, usually with a parent/guardian, will have weekly contact with the study dietitian, who will deliver interventions, review the diet and encourage adherence for Phase 1. The contact will be face to face at weeks 0, 1 and 4 and encouraged at weeks 2 and 3. However, during weeks 2 and 3, dependent on participant preference/commitments and the discretion of the dietitian, verbal support maybe given via phone or an electronic consult using Zoom, Skype, FaceTime or telehealth platforms approved by the health facility.

COVID 19: During the pandemic face to face visits will only be undertaken as directed by the health facility. At a minimum this will be at week 0 and 4 weeks. There will be no recruitment if the participants are unable to attend in person to the health facility.

It is anticipated that participants will find following the VLED protocol difficult, particularly during the first week. To address this, additional support will be provided by the study dietitian during week 1 via a phone call. The VLED protocol is based on current clinical practice at The Children's Hospital at Westmead. For the past 4 years it has been used with adolescent patients in the Weight Management Clinic, and was used in the SHAKE-IT pilot study of adolescents with obesity and type 2 diabetes[21].

The diet prescribed for Phase 1 of the study is a nutritionally complete VLED (Optifast® VLCD™, Nestlé Health Science, Nestlé Australia Ltd). Participants will choose to either consume:

1. Four Optifast® meal replacements per day (shakes and/or soups, and/or bars, and/or desserts), or
2. Three Optifast® meal replacements and one meal consisting of 100–150g lean, cooked meat and carbohydrate-free vegetables.

The diet is ~800 kcal/day, consisting of less than 40% of energy from carbohydrate (about 50 g/day), 40–55% of energy as protein and less than 20% of energy as fat.

To enhance compliance, participants will be given a list of foods considered to be energy-free that can be consumed ad libitum (Appendix 1) and the Optifast® meal replacements will be provided free of charge at each clinical study site. Participants will be encouraged to consume at least 2L of water or other energy-free beverages daily. At 4-5 weeks, participants will transition to one of the two hypocaloric diets.

Adherence to the VLED protocol

It is expected that some participants may not tolerate the VLED plan. All participants will be encouraged to continue the VLED for up to four weeks. To minimize drop outs, if the participant is not adhering to the VLED protocol, they will have the opportunity to transition early onto their prescribed diet. Early transitions will be recorded by the study dietitian.

Phase 2: Intensive diet intervention (weeks 5–16)

Participants will meet with the study dietitian at weeks 6, 9 and 12. Contact will be face-to-face, by phone or electronically using Zoom, Skype, Facetime or telehealth platforms approved by the health facility with at least one visit being a face-to-face meeting.

COVID 19: During the pandemic face to face visits will only be undertaken as directed by the health facility, and participants are able to meet with the study dietitian at weeks 6, 9 and 12 electronically using Zoom, Skype, Facetime or telehealth platforms approved by the health facility.

Additional support via text message, phone or email will be provided by the dietitian at weeks 8, 11 and 14. The dietitian will provide a prescribed meal plan and participants will choose and pay for their own food for the remainder of the intervention. The meal plans, titled 'My Meal Plan', will take into consideration adolescent food preferences with the aim of weight loss (see Appendix 2 and 3, sample meal plans). Given the high nutrient requirements for this age group and length of intervention, all participants will be given a commercial multivitamin (containing essential fatty acids) to ensure nutritional adequacy as used in previous studies [22]. A 'coaching' model will be used during consultations, which provides a theoretical basis for several psychological variables which are important in achieving dietary compliance, lifestyle change and sustainable weight loss, such as self-efficacy, stage of change intervention, autonomy, assuming responsibility, self-monitoring, goal setting, accountability and self-directed behaviour change [23].

a) Experimental arm (IER)

The IER protocol was developed from the FAST Diet Pilot Study (HREC/14/SCHN/189) and from Krista Varady's work in adults [17, 24] . It involves three energy restricted days each week, consuming one-third of daily energy requirements (~2520-2940 kJ or 600-700 kcal), and consuming a healthy diet for the four feeding days, referred to as 'regular eating' days. Participants will receive a meal plan containing detailed information on a wide variety of food choices that are appropriate for energy restricted and regular eating days. No energy restriction will be used on regular eating days, but participants will be given healthy eating guidelines, including recommended serves of fruit, vegetables and dairy food. These guidelines ensure nutritional adequacy. In addition, discretionary foods will be limited to one standard serve per week, and participants will have one 'meal off' per week incorporated into their plan to account for eating out and social gatherings.

b) Active control arm (standard hypocaloric diet)

The standard hypocaloric diet is high in fibre, with 40–50% of energy obtained from carbohydrate and 20-25% of energy from protein. The diet will have prescriptive energy levels based on age: 6000-7000 kJ (1430-1670kcal) for those aged 13-14 years or 7000–8000 kJ (1670–1900kcal) for those aged 15-17 years. Participants will receive detailed information on a variety of food choices. This is the current standard high-quality diet used in clinical weight management at The Children's Hospital at Westmead, and has been used previously in clinical trials including the RESIST study (HREC:07/CHW/12)[16] . The prescribed energy intake is not matched to the experimental arm. It is expected that participants allocated to the experimental arm will achieve average lower energy intakes, and therefore greater weight loss.

Phase 3: Step down diet intervention (weeks 17–52)

Participants in both the experimental arm (IER) and active control arm (standard hypocaloric diet) will continue with the prescribed intervention during this phase. The dietitian will review dietary intake and weight loss at each visit, modify goals as necessary, and encourage adherence to the intervention. During this phase, participants will have less contact with the study dietitian than in previous phases. Participants will meet with the dietitian at week 52 (end of study). At weeks 20, 26 and 36 the contact will be either face-to-face, by phone or electronically using Zoom, Skype,

Facetime or telehealth platforms approved by the health facility. A minimum of two contacts will be face-to-face during this time. Additional dietitian support will be provided via text message, phone or email at weeks 18, 24, 28, 42 and 48.

COVID 19: During the pandemic face to face visits will only be undertaken as directed by the health facility, and participants are able to meet with the study dietitian at weeks 20, 26 and 36 electronically using Zoom, Skype, Facetime or telehealth platforms approved by the health facility.

Goal weight
At baseline, dietitians will calculate the participants 'goal weight'. This goal weight will equate to an adult BMI of 25kg/m^2 using the International Obesity Federation Taskforce (IOTF) age and sex adjusted BMI cut-offs. Goal weights may be updated over the 12 month study period as values will change depending on age. Some participants may also have an *individual* goal weight. This weight is the target weight determined by discussion between the participant and dietitian. Individual goal weights are likely to be higher than the calculated goal weight but more realistic for some participants. Individual goal weights are not compulsory and not all participants may choose to define a specific goal weight. Both the calculated goal weight and individual weight loss goal should be documented in participant notes by the dietitian.

Weight maintenance
If a participant reaches their goal weight or their *individual* goal weight during the intervention period, they will have the option to transition to a weight maintenance style plan. This will be individualised according to the participants' allocated dietary pattern:

- *Intermittent energy restricted plan*: participants may transition from three energy restricted days to two, one or no days per week.
- *Hypo caloric plan*: The Australian Guide to Healthy Eating (2013) will be used.

Participants that are transitioned to a weight maintenance plan during the intervention period will be required to attend their subsequent dietetic review face-to-face, to ensure compliance with weight maintenance strategies.

Study Completion

At the week 52 appointment, during the final review with the study dietitian, an individualised plan for ongoing monitoring will be discussed and developed in collaboration with the participant and their family. All participants will be referred for ongoing care and follow-up to their referring physician, general practitioner, a local dietitian, or may remain within the weight management service provided by the hospital. Strategies for weight maintenance will be discussed and may include focusing on mealtime behaviours, increasing activity and self-monitoring (e.g. keeping a food and activity diary). Locally available services will be utilised where possible, e.g. phone coaching.

Physical activity, screen time and sleep

All participants will receive standard advice for physical activity, screen and sleep time as per the Australian Physical Activity & Sedentary Behaviour Guidelines for Young People (13-17 years) [25].

Psychological support

A psychologist will provide ongoing support to study staff for the duration of the 12 month study period. The psychologist will:

- Review all participant screening survey data
- Identify issues to discuss with study dietitians and paediatricians
- Assess participants who have been identified as 'at risk' by study clinicians or screening/monitoring surveys.

SMS support

In addition to the ongoing support provided by the dietitian during the study, participants will also receive planned weekly text messages. Messages will offer further advice, information, motivation and support. A bank of standardised, but personalised (e.g. participant's name) messages will support goals for diet, physical activity or sleep. Whilst most of the messages will apply to both study arms, some will be tailored to each dietary pattern. Please refer to Appendices 18 and 19 for details of the SMS messages and rationale behind the intervention. Participants who do not own a mobile will be asked to provide a suitable contact (e.g. parents' mobile number) to receive the text messages.

The standard SMS program used by The Children's Hospital at Westmead, provided by Message Media will be used to send messages to participants. This program is routinely used by The Children's Hospital at Westmead to communicate with patients (e.g. sending appointment reminders). Only authorised members of the research team will have access to participants' mobile numbers and the text messages. Dietitians at The Children's Hospital at Westmead will use this account to send appointment reminders and provide ongoing support to participants who wish to be contacted via text message. The Monash study site will use The Children's Hospital at Westmead's Message Media program to deliver the weekly SMS intervention to Melbourne based participants. However, this program will not be used for dietitian support or appointment reminders. The Monash team will instead use a study mobile. The myPace smartphone application may also be used for ongoing communications with participants. No personal information will be stored on the application. The dietitian will create a generic log in for each participant along with a unique code. Once the application is installed on the participant's smart phone or tablet, the participant will be asked to enter the code and will then have access to communicate with dietitians without providing any personal contact information.

It is not essential that participants receive the weekly text messages and participants will be given the opportunity to decline from receiving messages. They may opt out of receiving further messages at any time during the study. The number of participants who decide they do not want to receive the messages or those who opt out of receiving the messages during the intervention will be recorded.

Social Media support (Facebook)

Closed Facebook groups (private and invitation only) will be established for each study arm of the trial with the purpose of regularly sharing information relevant to participants. Participants will have the ability to comment on posts or post directly within the Facebook group. This will replace a traditional study newsletter.

Information will be posted to each group approximately once a week and may include:

- Recipes relevant to the study arm. For example, a 200 calorie dinner recipe for the IER group or low calorie substitutes/modified recipes for the reduced calorie group
- Ideas to encourage physical activity (e.g. tips for indoor activities on rainy days, promoting incidental activities such as taking the stairs)
- Tips for increasing physical activity during school holidays +/ or places to go in the area e.g. trampoline parks, rock climbing centres, bike tracks
- Tips and posts encouraging healthy eating during the school holidays or at birthday parties, friends' houses etc. (e.g. reminders about healthy, low calorie snacks)
- Local events such as team sports or cooking classes
- Sharing articles of interest

24 month follow up

At the final study appointment (week 52), participants and their parent/guardian will be asked if they agree to be contacted by the study team in 12 months (i.e. 24 months post-baseline), a consent form will be signed before contact is made. Contact will be maintained with families between 12 and 24 months. The study team will contact participants twice after study completion (6-8 weeks and 3-4 months after Week 52) to ensure referral services have been engaged. Participants will also receive a text message once every three months and on the participant's birthday. Participants who consent to be contacted 24-months post-baseline, will be invited to attend a single face-to-face appointment with a dietetic consultation and repeat the 12 month assessments (as detailed in Table 2). This is not an essential component of the study. A consent form will be signed for the 24-month appointment. Participants can opt out of receiving text messages at any time.

7.2.3 Intervention Fidelity

Background

Intervention Fidelity refers to the 'degree to which a program or intervention is implemented as intended by program developers' [26]. The fidelity of the intervention moderates the relationship between the study protocol and the observed outcomes [26].

An Intervention Fidelity evaluation substantiates study findings by confirming that the observed outcomes are secondary to the study protocol [26]. If an intervention has 'high fidelity', the outcomes can likely be attributed to measures implemented as part of the protocol. If a study is deemed to have 'low fidelity', we cannot verify that the outcomes are a consequence of the proposed protocol [27]. An understanding of the fidelity of an intervention ensures confidence when attributing outcomes to a protocol and supports the formulation of evidence based recommendations [26].

Interventions targeting lifestyle and behaviour change will often be adapted and will rarely run exactly as per the study protocol [27]. Intervention fidelity evaluations provide a careful account of how an intervention was adapted and consequently guide future attempts at successful implementation [27]. If an intervention is for larger scale dissemination, a fidelity evaluation will indicate the necessary processes required to consistently implement the intervention at a high level of quality, especially when implemented in different contexts with a range of facilitators [27].

The Medical Research Council United Kingdom (UK); Process evaluation of complex interventions guidelines [28], the National Institute for Health and Care Excellence UK Guidelines for Behavioural Change: Individual Approaches [29] and the National Institutes of Health USA Behaviour Change consortium [30] recommend a review of intervention fidelity as part of the study evaluation. Furthermore, the completion of an intervention fidelity evaluation will ensure that the Fast Track to Health protocol adheres with the Template for Intervention Description and Replication (TIDieR) reporting checklist for intervention studies [31].

How to measure Intervention Fidelity

Carroll et al. [26] propose a framework to measure intervention fidelity that considers both overall adherence of the study implementation to the original protocol and factors that may impact or moderate overall adherence to the protocol. The three key elements of this model include:

1. Measures of adherence

Measures of adherence assess whether an intervention adheres to the protocol as prescribed [26]. If an implemented intervention adheres to the content, frequency, duration, and coverage as outlined in the protocol, then fidelity can be said to be high [26].

2. Moderators of adherence

Moderators of adherence are factors that impact the capacity of an intervention to run as per the study protocol [26]. Potential moderators of study protocol include:

- Intervention complexity
- Facilitation strategies, including the provision of support to study facilitators
- Quality of delivery or the manner in which the intervention is delivered
- Participant responsiveness, including how engaged participants are by an intervention and their judgements as to the relevance of the program

3. Component analysis to identify 'essential' components

'Essential' components are determined during the evaluation phase of the intervention and are key to ensuring the adaptability of the intervention [26]. If the intervention is replicated, identifying the essential components of a protocol ensure that there is some flexibility in the study implementation as only these essential components, not the entire protocol, may need to be adhered to.

Measures of Adherence

Procedural Fidelity

Procedural fidelity assesses whether the intervention adheres to the protocol as prescribed [26]. The Fast Track to Health Dietitian Session Checklists outline the content to be discussed with participants during dietetic consultations at each specific time point. Study dietitians complete this checklist to report and record content discussed with participants during each consultation. To measure procedural fidelity, these self-reported checklists will be audited.

Dosage or Exposure to the Intervention

To measure participant exposure to the intervention, participant records on REDCap will be analyzed to assess;

- Attendance rates at face-to-face Dietitian sessions
- Total number of Dietetic sessions administered via telephone, online teleconferencing i.e. Skype or face time
- Total and type of messages sent via SMS as an additional support for participants

- Participant usage of the closed Facebook Groups

Moderators of Adherence

Facilitation strategies, quality of delivery and participant responsiveness will be measured to assess moderators of adherence to the study protocol.

Facilitation Strategies

Facilitation strategies refers to the support provided to clinicians to optimize and standardize intervention implementation [26]. Throughout the study, dietitians, study investigators and facilitators will keep a log of training provided or accessed throughout the study implementation. This log will be audited to review the types and frequency of support provision.

At the conclusion of the study, dietitians will be asked to complete a written questionnaire on what they believe is required to support future facilitators to implement this protocol if it is to be disseminated further (Appendix 21). Participation is optional, and questionnaires will be distributed electronically using Qualtrics. Dietitians will remain anonymous. The dietitian must read the participant information sheet and sign their informed consent prior to completing the written questionnaire.

Quality of Delivery

Quality of delivery refers to the manner in which the intervention is delivered and whether the style of delivery is appropriate to achieve the intended goals of the protocol [26]. Quality of Delivery will be measured using the Consultation and Relational Empathy (CARE) Measure (Appendix 22) [32]. The CARE Measure is a 10 question questionnaire developed for measuring rapport and empathy between clinicians and clients in an outpatient setting [32]. The questionnaire has been validated in adults [33] and the Visual CARE Measure is available for use with children (Appendix 23) [34]. Participants will be emailed the questionnaire via Redcap following the week 36 face-to-face Dietitian consultation. If the participant is unable to attend the week 36 consultation, the questionnaire will be sent for completion following the week 52 consultation. It will be at the discretion of the Dietitian as to whether they provide the participant with the Adult CARE measure or the Visual CARE Measure. The questions are consistent across both questionnaires, however the visual questionnaire contains additional images and graphics to support individuals with lower literacy skills [34]. Participation in the survey is optional.

Participant Responsiveness

Acceptability Questionnaires

Participant responsiveness will be evaluated using written acceptability questionnaires described in Section 7.5.

Semi-structured interview with participants

To further explore the acceptability of the intervention, participants and the parents or guardians of participants will be invited to participate in a semi-structured interview upon completion of the study. This interview will explore adolescent's and parent or guardians' opinions regarding the acceptability of the intervention. The questions will relate to three key themes including:

1. Overall acceptability and utility of the study recommendations.
2. Capacity to enact recommendations and the sustainability of the behaviour change.
3. Strategies to enhance adherence with the intervention.

Appendix 24 outlines a proposed script for the semi-structured interviews. The interviews will be conversational, and the interview facilitator may diverge from the proposed script to explore comments, concepts or ideas raised by the participant or their parent/guardian. The interview facilitator may slightly adjust questions to meet the needs of the participant. The scripts will consequently be used as a guide; however all questions will remain consistent with the outlined themes.

We intend to recruit a minimum of 16 participants with approximately 8 participants from both the Children Hospital at Westmead and Monash Children's Hospital. The sample will include participants from both study arms.

Parents/guardian and participants will be given an opportunity to complete the interview together or individually. At week 52 (i.e. study completion), participants and their parent/guardian will be provided with an information sheet outlining the purpose and structure of the interview. All participants and their parent/guardian must sign an informed consent form before participating. If participants choose to complete the interview alone they must sign an informed consent form along with their parent/guardian. Only the informed consent of the parent/guardian will be required if they choose to complete the interview without the adolescent present. A reminder email or text message will be sent to parents/guardians and/or participants one day prior to the scheduled interview time.

Semi-structured Interview with Dietitians

Following the completion of the study implementation phase, the study Dietitians will be invited to participate in a semi-structured interview regarding their observations of participant engagement and responsiveness. Questions will pertain to three key themes including:

1. The acceptability of the intervention for participants
2. Overall participant engagement and capacity to enact lifestyle recommendations
3. Observed barriers to participant and strategies which improved participant adherence to the study protocol

Participation in the interview is optional and no incentives will be provided to encourage participation in the interview. Study dietitians will be sent the participant information sheet via email once the majority of participants (>80%) have completed the study protocol. The email will encourage study dietitians to respond if they are interested in participating. A follow-up email will be sent after 7 days if there is no response to the initial email. If there is no response to the follow-up email, it will be assumed that the dietitian does not want to participate and no further attempts at recruitment will be made. The dietitian must read and sign their informed consent form prior to participating in the interview.

Facilitation of Semi-Structured Interviews

Interviews will be conducted face-to-face, via online video conferencing or telephone. Interviews will be conducted by a facilitator trained in the conduct of semi-structured interviews and is independent from the study implementation process. Interviews will be limited to a maximum of 60 minutes to prevent additional burden to interviewees although we expect interviews to take approximately 30-60 minutes. Participation is optional.

Semi-structured interviews have been chosen as the interview will be conversational and will allow the facilitator to explore the interviewee's opinions and experiences regarding the above themes. The interviews will be recorded and transcribed verbatim by a member of the study team. Participants will be de-identified during transcription of their interview.

Data Analysis

Synthesis of all quantitative data will be undertaken using SPSS. All qualitative data from written surveys and interviews will be managed, coded and analyzed using NVivo software. All qualitative data will be combined to capture any similar observations across different data collection techniques. An inductive thematic analysis will be used to analyze qualitative data.

Evaluation Outcomes

Proposed outcomes of this evaluation include:

1. Ensuring the Fast Track to Health protocol adheres to international recommendations for the evaluation of behavior change interventions
2. Data to support the evidence obtained as per the effectiveness and acceptability of the intervention
3. Reflective analysis of the study process to guide future attempts at implementation and dissemination of study findings
4. Analyzed and publishable quality evaluation data

7.2.4 Fitbit®

Participants who reach week 16 will be given a Fitbit® Flex 2 wristband to increase motivation and adherence to the intervention. Fitbit® activity monitors measure physical activity and sleep patterns. Various physical activities including running, outdoor bike riding, football, basketball, swimming and aerobic workouts can be tracked by the monitor, as well as simple steps. This is achieved using a 3 axis accelerometer which identifies the intensity of the activity being performed. The Fitbit® can also track sleep duration if worn to bed. Fitbit® devices have been shown to adequately measure both sleep and activity when compared to gold standard methods, however the accuracy and reliability of these measures is under examination [35, 36].

Participants' physical activity and sleep data will be accessed and downloaded by the study team. Data collection will only take place during week 16 and week 52, when participants are actively involved in the study. Participants who have been given a Fitbit® but withdraw from the study, will not have their data accessed after their withdrawal date. Participants will be encouraged to wear the Fitbit® during week 16 and week 52. Participants' data will be used for studies related to physical activity and sleep patterns. Fast Track dietitians will also access participants' Fitbit® accounts during diet reviews. For example, physical activity information may be used when discussing exercise or evaluating physical activity goals. Similarly, sleep data may be used by the dietitian when discussing sleep hygiene. Participants will be asked to create a Fitbit® account online or via the Fitbit® application. The study team and dietitian will log on to this account to access information recorded by the Fitbit®. This information is owned by the participant, therefore consent must be obtained before accessing and downloading the data.

A Fitbit® information sheet and consent form will be provided at week 16 to participants and their parent/guardian. A copy of the signed consent form will be given to participants and the hardcopy will be stored by the study team. It is not essential for this data to be collected, participants do not have to allow the study team to access their information, nor do they have to create an account to use the

Fitbit®. If participants do not want their information accessed, they will still be given a Fitbit®, which they can use as desired. Participants will not be required to return the Fitbit® at the conclusion of the study.

Account set up and security

To access information recorded by the Fitbit®, participants can create a Fitbit® account online or download the Fitbit® app to a smartphone or tablet. When creating their account, participants will be asked to provide an email address, date of birth, height, weight and gender. Some details will be used to provide individualized information to the user (e.g. calories burned). Participants will be asked to set a generic password, so the study team can access the account. At week 16, participants will be given instructions on how to create an account along with useful links to the Fitbit® website.

Once an account is created, participants may choose to add extra information such as food logs, alarms, and photos. All information recorded by the Fitbit® and entered into Fitbit® accounts is transferred to Fitbit® servers. Fitbit® servers are hosted and operated entirely in the United States, thus are subject to United States law. Fitbit® uses a combination of technical and administrative security controls to maintain the security of user's data. Identifiable information is not shared by Fitbit® unless under specific circumstances (e.g. compliance with the law). However, de-identified information may be shared or sold for research or reports about health and fitness, marketing and promotional use. When location features are active, Fitbit® collects data including GPS signals, device sensors and Wi-Fi access points to determine a user's specific location. This information is also stored on Fitbit® servers. Location features do not need to be active in order to use the Fitbit®. Additionally, if participants create an account using their smartphone, their contact list will be accessed to identify fellow Fitbit® users. This contact list is not stored and is immediately deleted. Participants will be informed that if they decide to use the Fitbit® and create an account, their physical activity, sleep information and any other information they've provided on their account will not be stored in Australia and does not come under Australian privacy laws
A complete outline of the Fitbit® privacy policy can be found at:
https://www.fitbit.com/au/legal/privacy.

Intervention resources

We have established protocols for all dietary interventions and the phone coaching sessions. Phone coaching and SMS content will be specific to the RCT study arm [7, 37].

7.2.5 Expected duration of the study

The overall study schedule is shown below in tables 1 and 2. It is anticipated that recruitment will require ~18 months.

Table 1: Study timeline

**Year**	**2017**			**2018**				**2019**				**2020**				**2021**				**2022**				**2023**			
Quarter	***2***	***3***	***4***	***1***	***2***	***3***	***4***	***1***	***2***	***3***	***4***	***1***	***2***	***3***	***4***	***1***	***2***	***3***	***4***	***1***	***2***	***3***	***4***	***1***	***2***	***3***	***4***
Planning, study set-up	■	■																									
Recruitment, baseline data			■	■	■	■	■	■	■	■	■	■	■	■	■												
Phase 1 int. (4 wk)			■	■	■	■	■	■	■	■	■	■	■	■	■												
Phase 2 int. (12 wk)				■	■	■	■	■	■	■	■	■	■	■	■	■											
Phase 3 int. (36 wk)				■	■	■	■	■	■	■	■	■	■	■	■	■	■	■	■								
4 mth outcomes complete																	■										
12 mth outcomes complete																			■								
24 mth outcomes complete																							■				
Data analysis, write up												■	■	■	■	■	■	■	■	■	■	■	■	■	■	■	■

Table 2 Schedule for enrolment, interventions and assessments

	STUDY PERIOD																								
	Enrolment	Allocation	Post-allocation																			Close-out			
TIMEPOINT**	$-t_1$	0	t_1	t_2	t_3	t_4	t_5	t_6	t_7	t_8	t_9	t_{10}	t_{11}	t_{12}	t_{13}	t_{14}	t_{15}	t_{16}	t_{17}	t_{18}	t_{19}	t_{20}	T_{21}		T_{22}
Weeks		0	*1*	*2*	*3*	*4*	*6*	*8*	*9*	*11*	*12*	*14*	*16*	*18*	*20*	*24*	*26*	*28*	*36*	*42*	*48*	52	*58-60*	*78*	*104*
**ELIGIBILITY AND CONSENT:**																									
**Screening Assessment [a]**	X																								
**Inclusion/Exclusion Criteria**	X																								
**Informed consent**	X																								X
**Informed assent**	X																								X
**Allocation**		X																							
**INTERVENTIONS:**																									
Phase 1 – jumpstart																									
Phase 2 – Intensive intervention																									
Phase 3 – Step-down diet																									
Dietitian face-to-face consult		X	X	X	X	X							X									X			X

***24 Month Follow Up*[b]**																									
***Dietitian 'other' consult*[c]**							X		X		X				X		X		X						
Dietitian support (phone, email, SMS)			X					X		X		X		X		X		X		X	X		X	X	
**ASSESSMENTS:**																									
***Weight*[d]**		X				X							X									X			x
***Height*[e]**		X				X							X									X			x
***Waist circumference*[f]**		X				X							X									X			x
***Bioelectrical impedance (BIA)*[g]**		X				X							X									X			x
***Dual energy x-ray (DXA)*[h]**		X											X									X			
***Psycho-behavioural parameters*[i]** *(Eating Disorder Examination Questionnaire (EDE-Q); Body Appreciation Scale; Weight Bias Internalisation scale; Binge Eating Scale; Rosenberg Self-Esteem Scale; Centre for Epidemiologic Studies Depression Scale Revised (CESDR-10); Weight on Quality of Life – Lite; Dutch Eating Behaviour Questionnaire (DEBQ); Depression Anxiety Stress Scale (DASS))*		X				X							X									X			x
***Diet assessment*[j]** *(Australian Child and*		X											X									X			x

Adolescent Eating Survey (ACAES))																									
Sedentary behaviours and physical activity assessment[i] *(Godin Leisure-time Questionnaire)*		X				X							X									X			x
Sleep[i] *(Pittsburgh Sleep Quality Index*		X				X							X									X			x
Blood pressure[j] *(systolic & diastolic)*		X				X							X									X			x
Blood sample[k] *(high-sensitivity CRP, total cholesterol triglycerides, HDL and LDL cholesterol, glucose and insulin levels, and liver function)*		X											X									X			x
Adherence[l] *(ketones)*			X	X	X	X							X									X			
Adherence[m] ***(diet recall)***		X									X								X						
Blood sample[n] ***(Monash only)*** *(Inflammatory markers, gene expression)*		X											X									X			
Blood sample[o] ***(CHW and Monash)*** *(later analyses)*		X											X									X			
Acceptability questionnaires						X							X									X			
Optional Resting Energy Expenditure (Monash only) & Visual Analog		X				X							X									X			

Intervention Fidelity: Consultation and Relational Empathy (CARE)																			X						
Intervention Fidelity: Semi structured interviews with participants and parents/guardians[r]																						X			

[a] Prior to enrolment, all participants will undergo a screening assessment to determine eligibility. This will include a clinical review either by a referring clinician or study doctor, assessment of biochemistry and/or blood glucose measurements via a finger prick blood sample using the Freestyle Optium Neo Blood Glucose and Ketone meter and completion of two screening questionnaires (EDE-Q, CESDR-10). This may also include assessment by a psychologist.
[b] A the Week 52 appointment, participants and their guardians will be offered the opportunity to attend a follow up visit at 24 months (i.e. 12 months after completion of the intervention).
[c] Dietitian ‘other’ consult may be either face-to-face, phone, or teleconference (Skype, zoom, FaceTime or telehealth platforms approved by the health facility), depending on participant preference and discretion of the dietitian. Between 4 and 16 weeks a minimum of one of the three visits will be face-to-face. Between 16 and 52 weeks, a minimum of two visits will be face-to-face between 16 and 52. COVID-19: During the pandemic face to face visits will only be undertaken as directed by the health facility
[d] Weight should be measured to the nearest 0.1kg by a blinded trained assessor on the electronic scales at each visit. At 24-months, participants will be asked to self-report their weight in an online questionnaire; those who attend an on-site appointment will also have their weight measured.
[e] Height should be measured to the nearest 0.1cm by a blinded trained assessor using a stadiometer at each visit. At 24-months, participants will be asked to self-report their height in an online questionnaire; those who attend an on-site appointment will also have their height measured.
[f] Waist circumference should be measured by a blinded trained assessor using a flexible steel tape measure to the nearest 0.1cm at the horizontal distance around the umbilicus using the left hand under technique.
[g] BIA should be measured by a blinded trained assessor on a Tanita MC780MA body composition analyser at The Children’s Hospital at Westmead and Seca 515 scales at BASE at Monash University at each visit.
[h] DXA should be measured by a trained assessor on the Prodigy, Lunar-GE DXA at the Children’s Hospital at Westmead site, and the GEiDXA at the Monash site.
[i] All questionnaires will be completed by participants on an iPad during the study visit. Details pertaining to each questionnaire can be found in Section 7.5 and attachments in the Appendix.
[j] Blood pressure should be measured by a blinded trained assessor using an automated monitor under standardized conditions.
[k] For these “standard blood tests”, a 20ml fasting blood sample will be collected by a blinded trained assessor using standard operating procedures. Blood samples will be stored in a secure -80°C freezer at the Monash site and then samples batched and processed later. At The Children’s Hospital at Westmead site the blood samples will be transferred to CHW Pathology for immediate processing.

[l] Ketones will be measured weekly during weeks 1-4 using blood from a finger prick using the Freestyle Optium Neo Blood Glucose and Ketone Monitoring System. Ketones will also be measured at week 16 and 52 for those in the IER group, using the Freestyle Optium Neo.
[m] Participants will be asked to complete the ASA24-Australia online dietary assessment tool prior to attending visits at baseline, week 12 and week 36 or will otherwise complete this on an iPad at the study site.
[n] 2ml of blood will be collected at the Monash site for testing of inflammatory markers and extraction of RNA if participants consent.
[o] At the Children's Hospital at Westmead a 10mL blood sample will be collected and stored in a -80°C freezer located at Westmead Biobank, in both serum and plasma aliquots, for later analyses. At the BASE facility at Monash University, 2mL of serum will be stored in a -80°C freezer for later analyses.
[p] At Monash only, resting energy expenditure (REE) will be measured by indirect calorimetry and will be used to assess the compensatory mechanisms associated with weight loss and REE. Two Visual Analog Scales will be used to rate feelings of hunger and satiety.
[q] Participants will be asked to complete an optional Consultation and Relational Empathy (CARE) questionnaire to evaluate quality of delivery as part of measuring Intervention Fidelity.
[r] Participants and their parents/guardians will have the option of completing a 30-60 minute interview as part of measuring Intervention Fidelity

7.2.6 Contingency plans

At each clinical visit, the investigator (or delegate) will determine whether any AEs have occurred. Adverse events will be recorded in the AE page of the eCRF. If known, the medical diagnosis of an AE should be recorded in preference to the listing of individual signs and symptoms. The investigator must follow up on the course of an AE until resolution or stabilisation. If an AE is ongoing after the end of study visit, the AE will continue to be followed up until resolution or stabilisation.

If, during the study period, a participant presents with a pre-existing condition that was not noted at the time of study entry, the condition should be retrospectively recorded in the Medical History section of the eCRF. For SAEs occurring during the study, the investigator or delegate will enter all relevant information in the AE page of the eCRF. AEs due to this low risk study are not expected to occur; none were observed in the pilot study. Where study results suggest patterns of adverse events, study investigators will discuss concerns with clinical teams in a timely manner.

7.2.7 Student involvement

This study will form part of a PhD project for Associate Investigator Ms Hiba Jebeile, who is an Accredited Practicing Dietitian enrolled at the University of Sydney. The student project will involve analysis of baseline data collected during the trial. We anticipate that other students will be involved in data collection, intervention delivery and data analysis at both sites.

7.3 Standard Care and Additional to Standard Care Procedures

Standard Care Procedures		
**Procedure**	**Time/ Visit**	**Volume**
Anthropometry	every visit	
Bioelectrical impedance analysis (BIA)	Baseline, weeks 4, 16, 52 and 24 months	
Biochemistry	Baseline, week 52 and 24 months	20ml of blood
Blood pressure	Baseline, weeks 4, 16,52 and 24 months	

Additional To Standard Care		
**Procedure**	**Time/ Visit**	**Volume**
DXA	Baseline, weeks 16 and 52	
Psycho-behavioural questionnaires	Baseline, weeks 4, 16, 52 and 24 months	
Biochemistry	Week 16	20mL of blood
Biochemistry	Baseline, weeks 16 and 52	10mL of blood for storage (CHW only)

Dietetic review*	Baseline, weeks 2, 4, 6, 9, 16, 26, 52, and 24 months		Dietetic review*	Weeks 1, 3, 6, 12, 20, 36, SMS/email/phone support and 24 months	
Clinical review	Baseline and 16 week		Psychologist Support	Baseline and as needed during 12 month study period	

* There is no single standard of care with these novel forms of dietary intervention. The indicated standard of care is based on current clinical practice at CHW.

7.4 Randomisation

After completion of screening visits, participants will be randomly allocated to one of the two intervention groups, using a computer-generated randomisation schedule (1:1) from the NHMRC Clinical Trials Centre (University of Sydney). Random allocation will occur via a process of minimisation with allocations concealed, and stratified by intervention site, age (13–14 years; 15–17 years), sex and body mass index (BMI) (equivalent to 30-34.9kg/m^2; 35-45 kg/m^2). Forced allocation will be used for participants who have a family member enrolled in the study to keep family members within the same study arm. The Interactive Voice Response System (IVRS) at the NHMRC Clinical Trials Centre is able to force the allocation of a new participant based on a previously randomised participant (based on the ID number). These new participants will be included in the stratification tables used to randomise subsequent participants. Assessors for the primary outcome (BMI z-score at 52 weeks) and secondary outcomes will be blinded to treatment allocation.

7.5 Study methodology

All testing and analysis involved in this study will be completed at The Children's Hospital at Westmead or the BASE Facility of Monash University. Procedures will be the same across sites, unless otherwise indicated. Participants will complete the following testing as part of the trial:

1. **Anthropometry:** Weight, height and waist circumference will be measured by an appropriately trained assessor blinded to treatment allocation, using standard protocols [38]. At 24-months participants will be asked to self-report their height and weight in an online questionnaire prior to their follow-up appointment; those who attend an on-site appointment will also have height and weight measured by a trained assessor.
2. **Bioelectrical impedance analysis:** Will be used to measure body composition**.** A stand-on Seca 515 scale will be used at Monash University and the Monash Children's Hospital and a Tanita MC780MA body composition analyser will be used at The Children's Hospital at Westmead.
3. **Dual energy x-ray absorptiometry (DXA):** This non-invasive procedure, with a very low-dose radiation exposure, will be used to measure body composition and fat mass. At The Children's Hospital at Westmead the Prodigy, Lunar-GE DXA (Madison, WI USA) will be used.

At Monash, the GE iDXA (Madison, WI, USA) will be used. Core scan software on the GE iDXA will be used to measure visceral fat in the Monash cohort.

4. **Psychological outcome questionnaires:** A range of psychological outcomes will be assessed using validated tools that are commonly used in this age group. Professor Susan Paxton will lead this component of the study. The questionnaires take approximately 30-40 minutes to complete on an iPad with REDCap survey software. The psychometric questionnaires that will be used are:

    - Eating Disorder Examination Questionnaire (EDE-Q) [39]
    - Body Appreciation Scale [40]
    - Weight Bias Internalisation scale [41]
    - Binge Eating Scale [42]
    - Rosenberg Self-Esteem Scale [43]
    - Centre for Epidemiologic Studies Depression Scale Revised (CESDR-10) [44]
    - Quality of Life using Impact of Weight on Quality of Life – Kids [45]
    - Dutch Eating Behaviour Questionnaire (DEBQ) [46]
    - Depression Anxiety Stress Scale (DASS-21) [47]

    The EDE-Q will be used as a screening tool at baseline to identify participants with an undiagnosed eating disorder requiring treatment prior to participation in a weight management study. Participant will be assessed and referred as per standard clinical practice.

    The CESDR-10 will be used as a screening tool at baseline to identify participants at high risk of clinical depression requiring referral for further assessment.

5. **Dietary assessment:** Diet quality, food choices and food patterns will be assessed using the Australian Child and Adolescent Eating Survey (ACAES)[48, 49], a validated self-administered, semi-quantitative food frequency questionnaire, which takes ~15 minutes to complete and provides immediate analysis of the results and comparison of food and nutrient intake with nutrition targets. Participants will complete the survey at baseline, week 16, week 52 and at 24-month follow-up. They will be sent a personalized link to the tool. Participants may complete the survey at home or on an iPad at their dietitian visits.
6. **Sedentary behaviour, physical activity and sleep quality**: will be assessed using validated tools appropriate for this age group. The questionnaires will be completed on an iPad using RedCap software. The questionnaires that will be used are:

    - Godin Leisure-time Questionnaire [50]
    - Pittsburgh Sleep Quality Index [51]

7. **Blood pressure:** Systolic and diastolic blood pressure will be measured using an automated monitor under standardised conditions by trained study personnel blinded to treatment allocation.
8. **Biochemistry:** A 20 mL fasting blood sample will be collected to measure high-sensitivity CRP, lipids (total triglycerides and high-density and low-density cholesterol), glucose and insulin levels, and liver function. As poor renal function is a contraindication for initiating a VLED plan,

urea and electrolytes (UEC) will be measured at baseline. All blood samples will be collected by trained personnel using standard operating procedures. The Children's Hospital at Westmead will also store a 10mL sample of blood for later analyses of a range of known (e.g. gut hormones and peptides (leptin and ghrelin) and metabolomics) and unknown (novel) biomarkers related to obesity and cardio-metabolic risk. These blood samples will be stored at Westmead Biobank. BASE at Monash University will store 2mL of the 20mL fasting blood samples as serum for later analyses of hormones and metabolites related to obesity.

9. **Inflammatory markers and RNA extraction:** 4 mL (from the 20ml) fasting blood sample will be stored within the Monash cohort to measure a range of inflammatory markers (adiponectin, HGF, IL-1β, IL-6, IL-8, leptin, MCP-1, NGF, PAI-1 (Total), resistin, TNF-α) and for RNA extraction. Blood samples will be collected by trained personnel using standard operating procedures.

10. **Adherence:** Ketones will be measured to assess adherence to the VLED protocol and IER plan. Blood ketones will be measured weekly during Phase 1, using the Freestyle Optium Neo Blood Glucose and Ketone Monitoring System. Ketones will also be measured using the Freestyle Optium Neo for the IER group only at week 16 and 52.

11. **Diet review:** Participants will be asked to complete the Automated Self-Administered 24 hour dietary assessment tool (ASA24-Australia) at baseline, week 12 and week 36. This will assess diet quality and adherence. Participants will be sent an individualized link to log into the assessment tool online. They may complete this in their own time at home or before their appointment with the dietitian. It will take approximately 30 minutes to complete. Additionally, weekly diet reviews will be conducted by the study dietitian until the end of phase 1, then reviews will reduce gradually throughout phases 2 and 3 (see Figure 2). Diet reviews will be used to assess adherence (24 hour recalls, as used in the RESIST study – HREC:07/CHW/12) [37], identify and address any problems following the diets, ensure adequacy of the diet and set goals for participant. As part of nutrition therapy, acceptability is monitored and support provided. The protocol is flexible and food preferences will be considered by the dietitian within the 'My Meal Plan' and Phase 3 Step-down diet intervention.

12. **Acceptability**: Participant views relating to acceptability of the diet intervention, program satisfaction and feedback will be collected in questionnaires at 4, 16 and 52 weeks. The questionnaires that will be used are:

    - Participant acceptability questionnaire

13. **Clinical assessment and review:** Prior to enrolment, all participants will have a clinical assessment to determine eligibility. This may be completed by a referring clinician or study doctor. A follow up medical review of participants by Junior or Senior Medical Officers at both hospitals will occur at 16 weeks unless otherwise indicated. Where participants are identified as requiring additional medical review outside of major outcome visits, these appointments should occur face-to-face.

14. **Psychological Assessment:** Some participants may require a review by the study psychologist. This will occur if the participant has been flagged as 'at risk' by study clinicians or screening/monitoring procedures and should occur face-to-face.

15. **Resting Energy Expenditure (Monash Only):** Resting energy expenditure (REE) will be measured by indirect calorimetry and will be used to assess the compensatory mechanisms associated with weight loss and REE. It will take approximately one hour to complete the measures. It will not be essential for participants to complete this assessment and will have the opportunity to decline participating in this procedure. Subjective feelings of hunger and satiety using Visual Analog Scales will be collected via an iPad prior to REE being measured. This takes two to three minutes for participants to complete.
16. **Intervention Fidelity:** Intervention fidelity will be evaluated using the framework developed by Carroll et al. [26]. Table 3 provides a summary of Methodology used for measuring Intervention Fidelity. Data will be collated and analysed quantitively using SPSS software.

17. **Gene Expression (Monash Only):** Gene expression will be measured by RNA sequencing and will be used to assess relationships between gene expression changes and weight loss. This investigation is of an exploratory nature. This analysis is optional for participants.

Assessments completed by parents

1. **Demographic:** Parents will be asked to complete a demographic questionnaire at baseline. The results of this will be used to describe the group profile of the Fast Track cohort. They will be asked to answer questions related to ethnicity, socioeconomic status and family medical history. Questions have been adapted from existing questionnaires [52-55]. It will not be essential for parents to answer all questions.

2. **Acceptability:** Parent views relating to the acceptability of the diet intervention, program satisfaction and feedback will be collected through questionnaires at 4, 16 and 52 weeks. The tool used to assess this will be:
    - Dietary intervention questionnaire (parents)

Table 3. Summary of methodology for measuring intervention fidelity

Implementation Fidelity Measure		Measure
Measures of adherence	Procedural fidelity	Audit of the Dietitian Framework Session Outline checklist
	Exposure or dose<br><br>(frequency/ duration)	• Attendance rates at face-to-face sessions<br>• Total exposure to additional supports via SMS/ telephone/ video teleconference<br>• Facebook data regarding use of the Facebook groups
Moderators of adherence	Intervention Complexity	• This intervention aims to address a multifactorial problem, comprises of multiple components and is being implemented across multiple sites. It would therefore be regarded as a complex intervention[28]
	Facilitation Strategies	• Audit of training log<br>• Dietitian Feedback Questionnaire

	Quality of delivery	• Completion of Consultation and Relational Empathy (CARE) Measure by Participants at week 36
	Participant responsiveness	• Acceptability questionnaires<br>• 30-60 minute semi-structured interviews with participants, parents/guardians as to practicality and acceptability of the intervention at completion (week 52).<br>• 30-60 minute semi-structured interviews with dietitians to evaluate the practicality and acceptability of intervention. Interviews will be scheduled based on the dietitian's availability.

7.6 Recruitment Procedure

7.6.1 Recruitment

Initially, recruitment will focus on internal referrals through hospital clinics at both The Children's Hospital at Westmead (Endocrinology and Weight Management clinics) and Monash. Following this initial recruitment, a range of strategies will be used including [56, 57] :

- Strong study branding and logo.
- Study website which will include study information for youth and parents, an expression of interest online form submission, direct phone and email contact details, links to other relevant resources and services.
- Information for patients on waiting lists at the participating clinics.
- A variety of communications to health professionals within the local area including GPs, paediatricians and dietitians.
- Youth-focused and parent-focused local media campaigns using media releases, radio, newspapers – via support of CHW and Monash PR Departments.
- Use of social media:
    - ➢ Existing Twitter feeds, institute and personal feeds (e.g. @MonashNutrition, @ProfTruby) may be used by research investigators in a professional manner to inform medical and health care professionals about the study and recruitment. For example 'Fast Track to Health, RCT of intermittent energy restriction vs. continuous energy restriction in adolescents with obesity NOW RECRUITING'
    - ➢ An active social media presence through a study page on Facebook will be established, containing basic information about the study with a primary objective of re-directing interested participants or parents to the study website. Interested participants or parents will be able to directly connect with the study through direct messaging from the Facebook page. The page will link directly to the Fast Track website where interested participants or parents will have the ability to complete an eligibility check and submit an expression of interest form.
    - ➢ The target audience for our Facebook page is: young people (13 – 17 years) living near the study site (either BASE at Monash University or The Children's Hospital at Westmead, parents of young people who may be eligible for the study and also health professionals who treat people who may be eligible for the study in the community.
    - ➢ To ensure our target audience is reached by the social media page, targeted Facebook ads will be used as a recruitment strategy:
        - o Participants: a Facebook ad will target participants within the geographic area of each study site. Ads will be targeted based on age (13-17yrs), postcode, and areas of interest including weight loss and dieting.
        - o Parents: a Facebook ad will target parents within the geographic area of each study site. Ads will be targeted based on age (35-55yrs), postcode, and areas of interest including parenting, weight loss and dieting.
        - o Evaluations will be completed to assess the spread and reach of the Facebook advertisements. Page views will also be evaluated by age, gender, device used etc., using the Facebook 'Insights' tab.

Young people who have participated in weight loss studies have often enquired about enrolment after having seen promotional materials several times or in different locations. We will offer a range

of ways of enquiring about the study: website, text messages, email or mobile. These will be monitored to determine the most successful recruitment strategies. Ethics approval will be sought prior to the implementation of each recruitment strategy.

7.6.2 Retention strategies

Strategies to retain participants in the study include:

- Thank you, birthday and Christmas/ New Year greetings.
- Facility for updating contact details and recording details of two family members or close friends.
- SMS reminders for appointments.

We will also provide:

- Some flexibility in scheduling appointments.
- At least weekly contact between the study team and participants offering a variety of contact methods including face-to-face meetings, Skype, face-time, zoom, telehealth appointments, telephone calls, emails, or SMS text messages, depending on the participants preference.
- Timely feedback on healthy lifestyle changes and weight goals using a practical, supportive and flexible approach suited to this age group.
- Free parking (Monash) or parking vouchers (CHW) and a petrol gift card at each face-to-face visit to assist with travel costs.
- Gift Vouchers at 52 week appointments ($20 Coles Myer gift card).
- Fitbit® wristbands (Fit Bit Flex 2) will be given to participants at week 16 to enhance motivation and engagement as contact with the dietitian decreases during the final phase of the study

7.7 Inclusion Criteria

- Adolescents aged 13–17 years (inclusive)
- Obesity (defined as equivalent to adult BMI 30–45 kg/m^2) [58] and <u>at least one</u> of the following metabolic complications:
  - o Pre diabetes: impaired fasting glucose 5.6-6.9mmol/L and/or impaired glucose tolerance 2 hour post load 7.8-11.1mmol/L
  - o Insulin resistance: fasting insulin (pmol/L)/glucose(mmol/L) ratio >20
  - o Presence of acanthosis nigricans
  - o Hypertension: systolic and/or diastolic blood pressure above the 90th percentile for sex, age and height
  - o Low HDL-C <1.03mmol/L
  - o High triglycerides ≥ 1.7mmol/L
  - o Abnormal Alanine Transaminase (ALT) or Gamma-Glutamyltransferase (GGT) based on standard PowerChart cut-points (i.e. ALT or GGT >45 U/L)
  - o Diagnosis of polycystic ovary syndrome (PCOS)

7.8 Exclusion Criteria

- Secondary obesity
- Significant intellectual disability as documented by the referring doctor
- Significant medical or psychiatric illness
- Previous diagnosis of Type 2 Diabetes Mellitus
- Currently undergoing treatment for a clinical eating disorder

- Pregnancy, or planning to become pregnant within the next 18 months
- Taking medications that have an effect on weight in the short term (excluding metformin)
- Adolescent or parent with poor level of spoken English
- Current enrolment in a weight loss program
- BMI in excess of 45kg/m^2

7.9 Screening Assessment

Screening assessments will be completed for all participants prior to enrolment. This may be completed as a standalone appointment or combined with the baseline visit.

The following must be done to confirm eligibility:

1. **Clinical assessment**: All participants will be assessed by a referring clinician or the study doctor.
2. **Biochemistry**: Fasting insulin, glucose, LFTs and lipids will be reviewed by the study doctor. At the on-site screening appointment all parents and adolescents will also be asked whether they consent to the adolescent's screening pathology results being used in analyses (irrespective of whether they are eligible to enrol).
3. **Blood Glucose Levels**: Trained personnel <u>may</u> also measure blood glucose levels via a finger prick blood sample using the Freestyle Optium Neo Blood Glucose and Ketone meter. This is to identify participants who may have undiagnosed Type 2 Diabetes. Participants will be excluded from the study if:
   a. Fasted blood glucose >7mmol/L
   b. Random blood glucose >11.1mmol/L
4. **Screening questionnaires**: The EDE-Q and CESDR-10 will be used to screen participants for eating disorders and depression. The questionnaires will be completed prior to enrolment in the study and are repeated at weeks 4, 16 and 52. Screening questionnaires are emailed to potential participants prior to attending an on-site appointment. At the on-site screening appointment all parents and adolescents will also be asked whether they consent to the adolescent's screening questionnaire data being used in analyses (irrespective of whether they are eligible to enrol). Should a participant complete screening questionnaires without attending an on-site appointment to provide consent, data collected will not be included in analyses.
5. **Psychological assessment**: A psychologist may be involved in participant assessment prior to enrolment. This will be based on screening questionnaires and input from the study doctors or dietitians.

Please note: If a recent blood test (taken in the previous 8 weeks) is available, this will be used to confirm eligibility. Alternatively, a fasting blood sample may be collected by trained personnel prior to a participant's enrolment in the study. This will include collection of bloods for storage. A consent form must be signed prior to the collection of fasting bloods. Stored blood samples collected for individuals who do not progress to enrolment will be destroyed immediately.

If the inclusion and exclusion criteria are met to the satisfaction of the reviewing practitioner/s, the participant will be enrolled into the study. Ineligible participants will be asked if they would like to be contacted by investigators in the future if suitable studies arise that may be of benefit to them.

7.10 Consent

The investigator is responsible for obtaining a participant's written informed consent to participate in the study from parent(s)/guardian(s). The Information Sheet and Consent Form prepared for the study complies with the provisions of ICH GCP and local legal requirements. To be able to engage in the consenting discussions, children <18 years of age will be provided with Information Sheet Only. Before undergoing screening procedures for possible enrolment into the study, participants/families must be informed, in an understandable form, about the nature, scope, and possible consequences of the study. This information must be given orally to participants by a physician or Accredited Practising Dietitian (according to applicable regulatory requirements) who is well informed about the nature, scope, and possible consequences of the study. Written information about the study will also be provided in a Participant Information Sheet. The date on which this oral and written information on the study was provided to the participant, and by whom it was provided, must be documented in the information Sheet and Consent Form.

8 Participant Safety and Withdrawal

8.1 Risk Management and Safety

There are no identified risks for participants as the study is relatively low risk with no invasive procedures and drug therapy. The study is being conducted by well trained and GCP accredited study personnel. Data generated from this study will only be presented as pooled results therefore, the confidentiality of participants will always be protected in dissemination of these research results. No individual participant will be able to be identified from publications and presentations arising from this study.

8.2 Adverse Event Reporting

8.2.1 Definitions

An adverse event is any undesirable sign, symptom or medical condition occurring after starting the study therapy (the IER diet). A medical condition/disease present before starting the study treatment will only be considered an adverse event if it worsens after starting the study treatment.

Pregnancy will be reported as an adverse event and followed to determine the outcome. Any study participant who becomes pregnant during the study will be withdrawn from the study.

A serious adverse event is an undesirable sign, symptom or medical condition which,

(1) is fatal or life-threatening,
(2) requires admission to hospital as an inpatient,
(3) results in persistent or significant disability/incapacity,
(4) constitutes a congenital anomaly or birth defect,
(5) is medically significant, may jeopardize the participant and may require medical or surgical intervention to prevent one of the outcomes listed above.

A SUSAR is a serious adverse event that is suspected to be related to the study but unexpected.

8.2.2 Assessment and Documentation of Adverse Events

Monitoring of relevant clinical and laboratory safety parameters will be undertaken by researchers at the time of each visit.

Any adverse event will be assessed and managed by the participant's physician, or general practitioner, with the event followed to resolution. Conditions that are present at screening and do not deteriorate will not be considered adverse events. Abnormal laboratory values will not be adverse events unless deemed clinically significant by the investigator and documented as such. Changes in the severity of an adverse event will be reported. Adverse events characterised as intermittent will be documented for each episode. All adverse events will be followed to adequate resolution, where possible.

Any serious adverse event will be reported in writing to the relevant HREC within 72 hours of the event. The description of each adverse event on the CRF will include:

- A description of the adverse event;
- The onset date, duration, date of resolution;
- Severity (mild, moderate or severe);
- Seriousness (i.e. is it an SAE?)
- Any action taken (e.g. treatment, follow-up tests);
- The outcome (recovery, death, continuing, worsening);
- The likelihood of the relationship of the adverse event to the study treatment (unrelated, possible, probable, definite).

The seriousness of an adverse event will be assessed by an investigator according to the definition stated above in this section, with the following exception:

Hospitalisation due to progression of disease will not be considered a serious adverse event for the purpose of this study.

Adverse events due to the study (rather than the clinical procedure) are not expected to occur. Where study results suggest patterns of adverse events related to clinical care, study personnel will discuss concerns with clinical teams in a timely manner.

An oversight committee will be responsible for the on-going monitoring of reports of serious adverse events (SAEs) as well as data relating to psychological outcome questionnaires. This committee will consist of independent clinical monitors, a statistician and trial investigator (non-voting role). They will be responsible for identifying safety concerns and make recommendations to the Trial Steering Committee for continuing or stopping the trial. The trial will be stopped if >5% participants overall are withdrawn by investigators for safety reasons related to the intervention. This will include any significant adverse events (SAEs) that occur.

8.2.3 Eliciting Adverse Event Information

All participants will be asked about any health related problems at each visit. Biochemical testing will occur as per the protocol. Adverse events will be recorded in case report forms.

8.3 Handling of Withdrawals

A participant may be withdrawn from the study if he/she becomes ineligible based on inclusion/ exclusion criteria e.g. becomes pregnant, develops an acute psychiatric problem.

Participants may withdraw from the study at any time. Those who withdraw will be encouraged to schedule a follow-up visit with their GP or physician.

8.4 Replacements

If participants withdraw they will not be replaced. Our sample size estimation accounts for a 30% attrition rate and we assume withdrawals will be equal between groups.

9 Statistical Methods

9.1 Sample Size Estimation & Justification

Our primary outcome is the difference in BMI z-scores between dietary intervention groups at 52 weeks. Based on our power calculations (see below) and assuming 30% attrition rate (which is consistent with attrition rates at 52 weeks of 23% for our RESIST study [16] and 30% for our Loozit study [7] we will recruit 186 adolescents; 124 at The Children's Hospital at Westmead; 62 at Monash) over an 18 month period.

Recruitment strategies are outlined above (please see 8.1.1 Recruitment).

The Children's Hospital Westmead

We will recruit 124 patients in total at The Children's Hospital at Westmead. We anticipate that we will recruit 40 from current hospital referrals and the remainder from the community. The Adolescent Weight Management Service provides a dietetic and medical clinic twice per month. The service currently receives 6-8 referrals per months on average, and has a waiting list of 12 months. Overall 6.1% of Australian adolescents are obese [1] which is higher in Western Sydney[59, 60]. Assuming the adolescent population in Western Sydney is ~270,000 [60] there will be at least 16,500 adolescents with obesity. We will need to recruit 0.5% of this population from the community.

Monash University and Monash Children's Hospital

We will recruit 62 patients in total at Monash. We anticipate we will recruit 50% from the existing Obesity Management Clinic at Monash Health under the direction of Dr Justin Brown and the others from generating new referrals from the community including local GPs and practice nurses. Pediatricians who consult at the newly opened Monash Children's Hospital will also be contacted in order to refer patients to the study who they see, but who they may not usually refer to an obesity service which is currently designed for complex conditions and those with co-morbidities.

9.2 Power Calculations

The Therapeutic Goods Administration (TGA) defines successful weight loss as at least a 5% decrease in initial bodyweight in adults. This is approximately equivalent to a 0.12 decrease in BMI z-score in adolescents. This intervention will be considered successful if there is a 5% difference between groups at 52 weeks as defined by the TGA [61].

Assuming a BMI z-score difference at 52 weeks of 0.12 and a SD 0.24 (based on our previous RCT of age and weight matched participants [61]; 80% power, and a two-sided significance level of 0.05, a sample size of 65 adolescents per group, 130 in total, is required. As indicated above we anticipate a 30% attrition rate and we will recruit 186 adolescents.

9.3 Statistical Methods to Be Undertaken

Data analyses will be carried out according to a pre–established plan, with the primary analysis based on intention to treat and with statistical support from the Biostatistician in the Office of Research, CHW. Analysis will be undertaken blinded. The difference in BMI z-score between groups at 52 weeks will be assessed using a t-test. Mixed models will also be used to investigate changes over time. The mixed models procedure will take into account within participant correlations and missing data points. A time by group interaction will be included to investigate whether rates of change of weight loss were significantly different between the two study groups. A/Prof Sarah Garnett along with SCHN statistician Elizabeth Barnes will oversee the statistical analyses.

Post hoc analysis will compare protocol adherers to non-adherers. An adherer will be defined as an individual who attends at least half of their scheduled appointments across the 12 month study period (either face to face or electronic). Additionally, at 52 weeks in measurement of the primary outcome (BMI z-score), a variation of plus or minus 4 weeks (i.e. measurement between 48 and 56 weeks) will be considered within protocol.

10 Storage of Blood and Tissue Samples

10.1 Details of where samples will be stored, and the type of consent for future use of samples

A blood sample will be taken from each participant at the start of the study, at week 16, 52 and 24-months. We will collect a fasting 20mL blood sample for immediate analysis of high-sensitivity CRP, lipids (total triglycerides and high-density and low-density cholesterol), glucose and insulin levels, and liver function. At baseline, week 16 and 52, at Monash University, 2 x 2mL of blood of the total 20ml will be stored as serum for future analyses of metabolites and hormones relating to obesity as well as inflammatory markers (plasma) and, if participants consent, gene expression analysis. Blood samples will be stored in a secure -80C freezer located at the Be Active Sleep Eat facility under participant code.

At baseline, week 16 and 52, at The Children's Hospital at Westmead, an additional 10mL blood sample will be collected from all participants and stored as plasma and serum for future analyses which may include metabolites and hormones relating to obesity. Stored blood samples will not be used for genetic testing. These blood samples will be stored in a secure -80C freezer located at Westmead Biobank until they are required for analysis.

11 Data Security & Handling

11.1 Details of where records will be kept & how long will they be stored

Information collected on participants for the purposes of the study will be stored as data on a secure online database (REDCap). Information will be identified by name, date of birth and email address for the purpose of study scheduling. After the completion of the study, all data will be converted into a coded, de-identified form prior to analysis. The Investigator at each clinical study site is responsible for ensuring that the source data are accurate, legible, contemporaneous, original and attributable, where the data are hand-written on paper, or entered electronically via online questionnaires or completion of electronic Case Report Form (eCRF).

REDCap will be kept on University of Sydney servers and is compliant with applicable laws and regulations governing the use of electronic records and/or electronic signatures.

Participants' names and nominated mobile contact will be stored on a secure online message service (Message Media) for the purpose of sending weekly SMS messages. This service is currently used by The Children's Hospital at Westmead to communicate with patients as standard practice.

Participants' sleep and physical activity patterns will be accessed and downloaded by the study team and stored securely on password protected files. Data will only be accessed by members of the study team at The Children's Hospital at Westmead and the Be Active Sleep Eat (BASE) facility at Monash University. After the study is complete, Fitbit® information will be stored for 15 years after which it will be destroyed.

The paper based source documentation would be certified copies which consist of a copy of the original information that has been verified as indicated by a dated signature, as an exact copy having all of the same attributes and information as the original. All paper-based documentation will be entered as electronic case report forms on REDCap

All the paper-based results with the study number of participants will be kept in a locked filing cabinet in the locked office of the Investigators at each clinical study site where security is in place.

At study completion, all identifiable study data will be treated as confidential and securely stored in accordance with Australian privacy laws for 15 years from the date of publication of results in both NSW and Victoria.

Data collected in hospital medical records will be retained by local study sites per data retention policies and procedures of that hospital.

11.2 Confidentiality and Security

All the participants will be given a code/study number in the study and the data will be identified using these codes. These anonymous codes will be used during all data analyses or presentations for future publications or conferences.

A master list of names and codes for re-identification will be kept separately in a spread sheet/database at local study sites. The Investigators at each clinical study site are the only people who can re-identify the data to each individual if necessary.

All documents, including clinical information pertaining to the study, biological samples and investigation results will be held in strict confidence by study investigators and not released to any third party without written approval of investigators and prior consent of participants. All participant information will be identifiable to allow for ongoing clinical care. However, study investigators will ensure participant confidentiality by storing all information in a password sensitive database. Access to data will be available only to investigators and the HREC where required.

11.3 Ancillary data

The study does not involve videos, photographs or images.

12 Appendix

Table 3: List of included attachments

**Document Name**	**Version Number, date**<br><br>**Date (e.g., 18 January 2012)**
Fast Track To Health Free Foods Resource	V2, 2017
FastTrack_Sample meal plan_IER	V2 2017
FastTrack_Sample meal plan_hypocaloric diet	V2 2017
Eating Disorder Examination –Questionnaire (EDE-Q)	V6.0, 2008
Body Appreciation Scale	2005
Weight Bias Internalisation scale	
Binge Eating Scale	1982
Rosenberg Self-Esteem Scale	
Centre for Epidemiologic Studies Depression Scale Revised (CESDR-10)	2004
Impact of Weight on Quality of Life – Kids	2002
Dutch Eating Behaviour Questionnaire	1986
Australian Child and Adolescent Eating Survey	2015
Godin Leisure-time Questionnaire	1997
Pittsburgh Sleep Quality Index	1988
Depression Anxiety Stress Scale-21	1995
Participant Acceptability Questionnaire Week 4	V1 2017
Participant Acceptability Questionnaire Week 16 + 52	V2, 2017
Parent Acceptability Questionnaire	V2, 2017
Fast Track Demographic Questionnaire	V1 2017

Fast Track SMS Messages	V1.2 2018
Fast Track SMS Rationale	V1 2017
Fast Track SMS Messages_12-24 months	V1, 2019
Fast Track Master Parent/Guardian Main Information Sheet and Consent Form	V2.7, 2019
Fast Track Master Participant Information Sheet	V2.5, 2018
Dietitian Feedback Questionnaire	V1, 2018
Consultation and Relational Empathy (CARE) Measure	
Visual Consultation and Relational Empathy (CARE) Measure	
Semi structured interview scripts	V1, 2018
Fast Track Master Fitbit Parent/Guardian Information Sheet and Consent Form	V1, 2018
Fast Track Master Participant Instructions for Fitbit	V1, 2018
Fast Track Master Evaluation Parent/Guardian Information Sheet and Consent Form	V1, 2018
Fast Track Master Evaluation Participant Information Sheet	V1, 2018
Fast Track Master Evaluation Dietitian Information Sheet and Consent Form	V1,2018
Fast Track Master Follow Up Parent/Guardian Information Sheet and Consent Form	V1.2, 2019
Fast Track Dietitian Session Checklists	
Visual Analog Scales	V1, 2019

13 References

1. Garnett, S.P., et al., *Trends in the Prevalence of Morbid and Severe Obesity in Australian Children Aged 7-15 Years, 1985-2012.* PLOS ONE, 2016. **11**(5): p. e0154879.
2. Garnett, S.P., et al., *Body mass index and waist circumference in midchildhood and adverse cardiovascular disease risk clustering in adolescence1,2,3.* The American Journal of Clinical Nutrition, 2007. **86**(3): p. 549.
3. Skinner, A.C., et al., *Cardiometabolic Risks and Severity of Obesity in Children and Young Adults.* New England Journal of Medicine, 2015. **373**(14): p. 1307-1317.
4. Ho, M., et al., *Effectiveness of lifestyle interventions in child obesity: A systematic review with meta-analysis.* Obesity Research & Clinical Practice, 2012. **6**: p. 54-55.
5. Oude Luttikhuis, H., et al., *Interventions for treating obesity in children.* Cochrane database of systematic reviews (Online), 2009(1): p. CD001872.
6. Ho, M., et al., *Effect of Fat Loss on Arterial Elasticity in Obese Adolescents With Clinical Insulin Resistance: RESIST Study.* The Journal of Clinical Endocrinology & Metabolism, 2014. **99**(10): p. E1846-E1853.
7. Nguyen, B., et al., *Twelve-Month Outcomes of the Loozit Randomized Controlled Trial: A Community-Based Healthy Lifestyle Program for Overweight and Obese Adolescents.* Archives of Pediatrics & Adolescent Medicine, 2012. **166**(2): p. 170-177.
8. Truby, H., et al., *Adolescents seeking weight management: Who is putting their hand up and what are they looking for?* Journal of Paediatrics and Child Health, 2011. **47**(1-2): p. 2-4.
9. Ho, M., et al., *Effect of a prescriptive dietary intervention on psychological dimensions of eating behavior in obese adolescents.* INTERNATIONAL JOURNAL OF BEHAVIORAL NUTRITION AND PHYSICAL ACTIVITY, 2013. **10**: p. 119.
10. Gow, M.L., *Type 2 diabetes in children and adolescents: prevention and treatment by lifestyle intervention*. 2016, Discipline of Paediatrics and Child Health
11. Collins, C.E., et al., *12Month changes in dietary intake of adolescent girls attending schools in low-income communities following the NEAT Girls cluster randomized controlled trial.* Appetite, 2014. **73**: p. 147-155.
12. Kornman, K.P., et al., *Electronic therapeutic contact for adolescent weight management: The Loozit® study.* Telemedicine and e-Health, 2010. **16**(6): p. 678-685.

13. Gow, M.L., et al., *Youth with type 2 diabetes who adhere to a very low energy diet achieve rapid weight loss and remission of type 2 diabetes; the SHAKE IT pilot study*, in *Australian & New Zealand Obesity Society 2015 Annual Scientific Meeting*. 2015: Melbourne.
14. Gow, M.L., et al., *Impact of dietary macronutrient distribution on BMI and cardiometabolic outcomes in overweight and obese children and adolescents: a systematic review.* Nutrition Reviews, 2014. **72**(7): p. 453-470.
15. Collins, C., et al.,, *DAA Best Practice Guidelines for the Treatment of Overweight and Obesity in Adults; Report to inform the 2011 revision of the 2005 guidelines* 2011, Dietitians Association of Australia.
16. Garnett, S.P., et al., *Optimal Macronutrient Content of the Diet for Adolescents With Prediabetes; RESIST a Randomised Control Trial.* The Journal of Clinical Endocrinology & Metabolism, 2013. **98**(5): p. 2116-2125.
17. Varady, K.A., et al., *Short-term modified alternate-day fasting: A novel dietary strategy for weight loss and cardioprotection in obese adults.* American Journal of Clinical Nutrition, 2009. **90**(5): p. 1138-1143.
18. Klempel, M.C., et al., *Dietary and physical activity adaptations to alternate day modified fasting: Implications for optimal weight loss.* Nutrition Journal, 2010. **9**(1): p. 35-35.
19. Doolen, J., P.T. Alpert, and S.K. Miller, *Parental disconnect between perceived and actual weight status of children: A metasynthesis of the current research.* Journal of the American Academy of Nurse Practitioners, 2009. **21**(3): p. 160-166.
20. Ramos-Lopez, O., et al., *Guide for current nutrigenetic, nutrigenomic, and nutriepigenetic approaches for precision nutrition involving the prevention and management of chronic diseases associated with obesity.* Lifestyle Genomics, 2017. **10**(1-2): p. 43-62.
21. Gow, M.L., et al., *Reversal of type 2 diabetes in youth who adhere to a very-low-energy diet: a pilot study.* Diabetologia, 2017. **60**(3): p. 406-415.
22. Lister, N.B., et al., *Nutritional adequacy of diets for adolescents with overweight and obesity: considerations for dietetic practice.* EUROPEAN JOURNAL OF CLINICAL NUTRITION, 2017. **71**(5): p. 646-651.
23. Elfhag, K. and S. Rössner, *Who succeeds in maintaining weight loss? A conceptual review of factors associated with weight loss maintenance and weight regain.* Obesity Reviews, 2005. **6**(1): p. 67-85.

24. Hoddy, K.K., et al., *Meal timing during alternate day fasting: Impact on body weight and cardiovascular disease risk in obese adults.* Obesity, 2014. **22**(12): p. 2524-2531.
25. *Make your move – Sit less, Be active for life! Australian Physical Activity and Sedentary Behaviour Guidelines, 13-17 years*, D.o.H. Australian Government, Editor. 2014.
26. Carroll C, P.M., Wood S, Booth A, Rick J, Balain S., *A conceptual framework for implementation fidelity.* Implement Sci, 2007. **2**(40).
27. Breitenstein SM, G.D., Garvey CA, Hill C, Fogg L, Resnick B., *Implementation fidelity in community-based interventions.* Res Nurs Health, 2010. **33**(2): p. 164-173.
28. Moore GF, A.S., Barker M, Bond L, Bonell C, Hardeman W, et al. , *Process evaluation of complex interventions: Medical Research Council guidance.* BMJ : British Medical Journal, 2015. **350**(h1258).
29. NICE, *Behaviour change: individual approaches*. 2013, United Kingdom: National Institute for Health and Care Excellence.
30. Bellg AJ, B.B., Resnick B, Hecht J, Minicucci DS, Ory M, et al. , *Enhancing treatment fidelity in health behavior change studies: best practices and recommendations from the NIH Behavior Change Consortium.* Health Psychol, 2004. **23**(5): p. 443-451.
31. Hoffmann TC, G.P., Boutron I, Milne R, Perera R, Moher D, et al., *Better reporting of interventions: template for intervention description and replication (TIDieR) checklist and guide.* BMJ : British Medical Journal, 2014. **348**(g1687).
32. Heaney D, M.M., Mercer S, Watt G. , *The consultation and relational empathy (CARE) measure: development and preliminary validation and reliability of an empathy-based consultation process measure. .* Family Practice, 2004. **21**(6): p. 669-705.
33. Heaney, D., et al., *The consultation and relational empathy (CARE) measure: development and preliminary validation and reliability of an empathy-based consultation process measure.* Family Practice, 2004. **21**(6): p. 699-705.
34. Place MA, M.J., Duncan EAS, Reid JM, Mercer SW. 2016;20(1):55-67., *A preliminary evaluation of the Visual CARE Measure for use by Allied Health Professionals with children and their parents. .* Journal of Child Health Care., 2016. **20**(1): p. 55-67.
35. Zambotti M, B.F., Willoughby AR, Godino JG, Wing D, Patrick K, Colrain IM. , *Measures of sleep and cardiac functioning during sleep using a multi-sensory commercially available wristband in adolescents.* Physiology & Behaviour, 2016. **158**: p. 143-149.
36. Tully MA, M.C., Heron L, Hunter RF. , *The validation of Fitbit Zip physical activity monitor as a measure of free-living physical activity.* BMC Research Notes, 2014. **7**(952): p. 1-5.

37. Garnett, S.P., et al., *Researching effective strategies to improve insulin sensitivity in children and teenagers - RESIST. A randomised control trial investigating the effects of two different diets on insulin sensitivity in young people with insulin resistance and/or pre-diabetes.* BMC Public Health, 2010. **10**(1): p. 575-575.
38. Norton, K. and T. Olds, *Anthropometrica*. 1996, Sydney: University of New South Wales Press.
39. Fairburn, C.G., et al., *Assessment of eating disorders: Interview or self-report questionnaire?* International Journal of Eating Disorders, 1994. **16**(4): p. 363-370.
40. Avalos, L., T.L. Tylka, and N. Wood-Barcalow, *The Body Appreciation Scale: Development and psychometric evaluation.* Body Image, 2005. **2**(3): p. 285-297.
41. Durso, L.E. and J.D. Latner, *Understanding Self-directed Stigma: Development of the Weight Bias Internalization Scale.* Obesity, 2008. **16**(S2): p. S80-S86.
42. Gormally, J., et al., *The assessment of binge eating severity among obese persons.* Addictive Behaviors, 1982. **7**(1): p. 47-55.
43. Rosenberg, M., *Society and the adolescent self-image*. 1965, Princeton, N.J U6 Princeton University Press.
44. Haroz, E.E., M.L. Ybarra, and W.W. Eaton, *Psychometric evaluation of a self-report scale to measure adolescent depression: The CESDR-10 in two national adolescent samples in the United States.* JOURNAL OF AFFECTIVE DISORDERS, 2014. **158**: p. 154-160.
45. Brazier, J.E., et al., *Estimating a Preference-Based Single Index for the Impact of Weight on Quality of Life-Lite (IWQOL-Lite) Instrument from the SF-6D.* Value in Health, 2004. **7**(4): p. 490-498.
46. van Strien, T., et al., *The Dutch eating behavior questionnaire (DEBQ) for assessment of restrained, emotional, and external eating behavior.* International Journal of Eating Disorders, 1986. **5**(2): p. 295-315.
47. Lovibond, S.H.L., P.F. , *Depression, Anxiety and Stress Scale - 21 items (DASS-21)*. Manual for the Depression Anxiety & Stress Scale 2nd edition. 1995: Psychology Foundaiton.
48. Watson, J.F., et al., *Reproducibility and comparative validity of a food frequency questionnaire for Australian children and adolescents.* International Journal of Behavioral Nutrition and Physical Activity, 2009. **6**(1): p. 62-62.
49. Burrows, T., et al., *A comparative validation of a child food frequency questionnaire using red blood cell membrane fatty acids.* European Journal of Clinical Nutrition, 2012. **66**(7): p. 825-829.
50. *Godin Leisure-Time Exercise Questionnaire.* Medicine&amp Science in Sports &amp Exercise, 1997. **29**(Supplement): p. 36-38.

51. Buysse, D.J., et al., *The Pittsburgh sleep quality index: A new instrument for psychiatric practice and research.* Psychiatry Research, 1989. **28**(2): p. 193-213.
52. Evidence, C.f.E.a., *Population Health Survey 2012 Questionnaire*, C.f.E.a.E.-N.M.o. Health, Editor. 2012, NSW Health. p. 1-56.
53. *FAST Baseline demographic_v2.0*. 2016, The Children's Hospital at Westmead.
54. Institute, B.H.D., *AusDiab General Questionnaire 04/05.* 2005: p. 1-13.
55. Cancer Council, H.F., *National Secondary Students' Diet & Activity Survey*. 2013.
56. Nguyen, B., et al., *Recruitment challenges and recommendations for adolescent obesity trials.* Journal of Paediatrics and Child Health, 2012. **48**(1): p. 38-43.
57. Partridge, S.R., et al., *Effective Strategies to Recruit Young Adults Into the TXT2BFiT mHealth Randomized Controlled Trial for Weight Gain Prevention.* JMIR research protocols, 2015. **4**(2): p. e66.
58. Cole, T.J., et al., *Establishing a standard definition for child overweight and obesity worldwide: international survey.* BMJ : British Medical Journal, 2000. **320**(7244): p. 1A0-1240.
59. Hardy, L.L., et al., *30-year trends in overweight, obesity and waist-to-height ratio by socioeconomic status in Australian children, 1985 to 2015.* Int J Obes, 2017. **41**(1): p. 76-82.
60. Capuano, G., *Western Sydney profile – a region of diversity and growth*, in*.id the population experts*. 2015: .id Consulting Pty Ltd.
61. Garnett, S.P., et al., *Improved insulin sensitivity and body composition, irrespective of macronutrient intake, after a 12 month intervention in adolescents with pre-diabetes; RESIST a randomised control trial.* BMC PEDIATRICS, 2014. **14**(1): p. 289.
