# Supplementary material for: Symptoms of Depression, Eating Disorders, and Binge Eating in Adolescents With Obesity: The Fast Track to Health Randomized Clinical Trial
Source: JAMA Pediatr. 2024 Aug 26:e242851. Online ahead of print. doi: 10.1001/jamapediatrics.2024.2851 (PMC11348093; doi:10.1001/jamapediatrics.2024.2851)
Supplement: Supplement 2. — Trial Protocol Amendments. [file jamapediatr-e242851-s002.pdf]

## Protocol changes since commencement of the study

| Version Number | Modifications | Date of HREC approval |
|---|---|---|
| 2 | • Addition of support phone call during week-1<br>• Addition of method for measuring resting energy expenditure (REE) at Monash | 31.10.17<br>*(Prior to first participant enrolment)* |
| 2.1 | • Removal of blood collection at week-4<br>• Addition of very low energy diet acceptability questionnaire at week-4 | 20.2.18<br>*(before first participant reached Week 4)* |
| 2.2 | • Additional REE measurement added at week-4<br>• Provision of Fitbit at week-16 | 21.5.18 |
| 2.3 | • Addition of intervention fidelity protocol (including use of CARE questionnaire and semi-structured interviews with participants and parents/guardians) (PhD sub-study)<br>• Addition of weight maintenance procedures for each study arm for participants who reach their goal weight | 17.10.18 |
| 2.4 | • Addition of participant follow-up at 24 months (including additional consent to contact families at 24 months) | 30.01.19 |
| 2.5 | • Inclusion of clinical psychologist support including for review of screening surveys, liaising with other study clinicians and clinical assessment of 'at risk' participants<br>• Inclusion criteria updated to include requirement for participants to have at least one cardiometabolic complication | 03.04.19 |
| 2.6 | • Addition of gene expression analysis at Monash (PhD sub-study)<br>• Details of participant management at study completed added, including engagement of referral services and transition to weight maintenance | 30.07.19 |
| 2.9 | • Details of the study Oversight Committee added*<br>**This committee consisted of independent clinical monitors, a statistician and trial investigator (non-voting role). They were responsible for identifying safety concerns and making recommendations to the Trial Steering Committee for continuing or stopping the trial. The trial would be stopped if >5% participants overall were withdrawn by investigators for safety reasons related to the intervention. This would include any significant adverse events (SAEs) that occur.* | 12.02.20 |
| 3 | • Full 24-month follow-up procedures added, including the option for on-site review and/or completion of online questionnaires | 14.04.20 |

| | | |
|---|---|---|
| 3.1 | • Use of telehealth platforms (previously limited to Zoom, Skype and FaceTime) updated to include platforms approved by the health facility.<br>• COVID-19 clause added to specify that face-to-face appointments will occur in line with hospital directives. | 13.09.21 |
| 3.2 | • Procedures were updated to stipulate that appointments following the transition to weight maintenance or any additional reviews (for clinical or psychological reasons) should occur face-to-face and not via telehealth | 28.07.22 |
