# Supplementary material for: Symptoms of Depression, Eating Disorders, and Binge Eating in Adolescents With Obesity: The Fast Track to Health Randomized Clinical Trial
Source: JAMA Pediatr. 2024 Aug 26:e242851. Online ahead of print. doi: 10.1001/jamapediatrics.2024.2851 (PMC11348093; doi:10.1001/jamapediatrics.2024.2851)
Supplement: Supplement 3. — Statistical Analysis Plan. [file jamapediatr-e242851-s003.pdf]

Fast Track Statistical Analysis Plan

Sample size calculations

The Australian Therapeutic Goods Administration (TGA) defines successful weight loss as at least a 5% decrease in initial bodyweight in adults. This is approximately equivalent to a 0.12 decrease in BMI z-score in adolescents. This intervention will be considered successful if there is a 5% difference between groups at 52 weeks as defined by the TGA.

The sample size is based on the primary outcome, change in BMI z-score between dietary intervention groups at 52 weeks. Assuming a difference in the change in BMI z-score at 52 weeks of 0.12 and a SD 0.24 (based on our previous RCT which also recruited adolescents with obesity), 80% power, and a two-sided significance level of 0.05, a sample size of 65 adolescents per group, 130 in total, is required. Assuming a 30% attrition rate, which is consistent with attrition rates of 23% to 30% from our previous studies, we will recruit 186 adolescents.

Data cleaning

Data will be exported and cleaned, blinded to treatment allocation, according to the *Fast Track SOP for data export and cleaning*. Each step of data cleaning will be recorded in the data cleaning decision log. Data cleaning will include checks of variable names, labels and type and that all participant data is captured. Box plots, histograms, Q-Q plots, other tests (Kolmogorov-Smirnov, Shapiro-Wilk) will be used to ascertain normality for continuous variables. Missing values and outliers will be identified and reviewed.

Questionnaire sub-scale and global scores will be calculated, accounting for missing data in calculation of scores. Patterns of missing responses to questionnaires will be reviewed. Where scores are a sum of questions, mean imputation will be used to account for missing responses. Where >30% of responses are missing for one questionnaire, this timepoint will be excluded.

Data analysis

IBM SPSS Statistical Software, version 28 (IBM), will be used for data analysis. All analyses will be conducted blinded to group allocation. Data will be assessed for normality and nonparametric tests will be used as appropriate.

We will use intention-to-treat analysis and all participant data will be retained. Primary analysis will be linear mixed models, with an autoregressive first order covariance structure and restricted maximum likelihood (REML), will be used to estimate the change in outcomes between baseline, 4, 16, and 52 weeks. A pairwise post-hoc comparison will be conducted to compare means over time. Results will be presented as the difference in estimated marginal means (EMMs) ± 95%CIs. For the primary outcome, completer analysis will be conducted to estimate the mean difference ± SD between baseline and 52 wk using a paired t test for normally distributed data, or Wilcoxon's Signed Rank test for nonparametric data. Completer analysis will be reported when results are different to those from linear mixed models. Differences of $P \leq 0.05$ were considered statistically significant.
