# Supplementary material for: Symptoms of Depression, Eating Disorders, and Binge Eating in Adolescents With Obesity: The Fast Track to Health Randomized Clinical Trial
Source: JAMA Pediatr. 2024 Aug 26:e242851. Online ahead of print. doi: 10.1001/jamapediatrics.2024.2851 (PMC11348093; doi:10.1001/jamapediatrics.2024.2851)
Supplement: Supplement 4. — eMethods. Screening eTable 1. The Center for Epidemiologic Studies Depression Scale–Revised 10-Item Version for Adolescents Cut Points Based on the Scoring Described by Haroz et al eFigure 1. CONSORT Figure eTable 2. Proportion of Adolescents With Symptoms of Depression, Eating Disorders, and Binge Eating and Change Over Time by Group eFigure 2. Proportion of Adolescents Meeting Depression Screening Criteria Over Time, All Participants Combined eFigure 3. Proportion of Adolescents Meeting Eating Disorder Screening Criteria Over Time, All Participants Combined eFigure 4. Proportion of Adolescents Meeting Binge Eating Scale Criteria Over Time, All Participants Combined eReferences [file jamapediatr-e242851-s004.pdf]

Supplementary Online Content

Jebeile H, Baur LA, Kwok C, et al. Symptoms of depression, eating disorders, and binge eating in adolescents with obesity: the Fast Track to Health randomized clinical trial. *JAMA Pediatr*. Published online August 26, 2024. doi:10.1001/jamapediatrics.2024.2851

eMethods. Screening

eTable 1. The Center for Epidemiologic Studies Depression Scale–Revised 10-Item Version for Adolescents Cut Points Based on the Scoring Described by Haroz et al

eFigure 1. CONSORT Figure

eTable 2. Proportion of Adolescents With Symptoms of Depression, Eating Disorders, and Binge Eating and Change Over Time by Group

eFigure 2. Proportion of Adolescents Meeting Depression Screening Criteria Over Time, All Participants Combined

eFigure 3. Proportion of Adolescents Meeting Eating Disorder Screening Criteria Over Time, All Participants Combined

eFigure 4. Proportion of Adolescents Meeting Binge Eating Scale Criteria Over Time, All Participants Combined

eReferences

This supplementary material has been provided by the authors to give readers additional information about their work.

© 2024 Jebeile H et al. *JAMA Pediatrics*.

eMethod: Screening

Measures

Symptoms of depression

The CESDR-10 is a self-report measure of symptoms of depression adapted from the Centre for Epidemiological Studies Revised 20-item instrument[1] by Haroz et al.[2] The CESDR-10 has been shown to have good psychometric properties as a screening tool to identify adolescents who are at risk of having undiagnosed clinical depression across two nationally representative samples of adolescents (sample 1, n = 3777, age 13 to 18 years; sample 2, n = 1150, 10 to 15 years) in the United States. Internal consistency reliability was high in both samples (Cronbach's alpha (α) = 0.91 for sample 1; α = 0.90 for sample 2).[2]

Each item is rated on a 5-point ordinal scale from 0=not at all or less than 1 day in the last week; 1=1–2 days in the last week; 2=3–4 days in the last week; to 3=5–7 days in the last week or 3=nearly every day for 2 weeks[2]. Scores on each item are summed to provide a total scale on which higher scores indicate more symptoms of depression. CESDR-10 cut-points (**eTable 1**) were set based on the scoring criteria described by Haroz et al.[2] This classifies symptoms of depression as being sub-threshold, or a possible, probable or major depressive episode. As the expected prevalence of depression within samples of adolescents seeking obesity treatment is unknown, the lowest categorisation of a *possible* major depressive episode was used. This allowed all adolescents at risk of having depression to undergo a clinical assessment. A cut-point of 8 was used to identify the presence of any symptoms of depression in analyses.

eTable 1: The Centre for Epidemiologic Studies Depression Scale- revised 10-item version for adolescents cut-points based on the scoring described by Haroz et al.

Classification	Definition
**Major depressive episode**	The presence of anhedonia, dysphoria, OR irritability (score = 3) AND at least 4 additional symptoms with a score of 3
**Probable major depressive episode**	Defined as the presence of anhedonia, dysphoria OR irritability (score = 3) AND an additional 3 symptoms with a score of 3
**Possible major depressive episode**	Defined as the presence of anhedonia, dysphoria or irritability (score of 3) AND an additional 2 symptoms with a score of 3
**Subthreshold depression symptoms**	score ≥ 8, but do not meet above criteria
**No clinical significance**	score < 8

Eating disorder symptoms

The EDE-Q[3] is a self-report measure which can be used as a screening tool to identify participants at risk of having an undiagnosed eating disorder. The EDE-Q has acceptable validity in detection of disordered eating behaviours in adolescents with obesity (sensitivity=0.57-0.79, specificity=0.68-1.0, positive predictive value=0.22-1.0)[4-6], and is a superior screening tool for young adult women for the identification of disordered eating pathology in a primary care setting[7]. Higher scores on the EDE-Q indicate the presence of more disordered eating pathology and behaviours.

A 28-item version of the EDE-Q 6.0 was used, with questions relating to self-reported height, weight and menstruation excluded as these were captured during investigator-led assessments. Each item is rated on a seven-point scale, with scores of zero (no days in the past 28 days) to six (every day in the past 28 days). Scores on the four subscales (eating concern, weight concern, shape concern, dietary restraint) are determined by summing responses to each subscale item divided by the number of items included within the subscale, resulting in a maximum score of six. The global score is the average of the four subscale scores. Disordered eating behaviours (overeating, loss of control, binge eating [overeating with loss of control], self-induced vomiting, laxative use) are captured by asking participants to record the number of occurrences of the behaviour within the last 28 days.

© 2024 Jebeile H et al. *JAMA Pediatrics*.

In the absence of published norms for the EDE-Q for adolescents with obesity, an EDE-Q cut-point score of 2.7 for adolescents (16-19.9years), with no BMI criterion, as suggested by Ro et al.,[8] was used. In addition to a cut-point, the protocol included the identification of adolescents who reported any purging behaviour or who reported two or more episodes of overeating, loss of control or binge eating (overeating with loss of control) in the previous 28 days. While only the question relating to purging behaviours has been shown to have acceptable validity,[9] the additional behaviours were included as part of the screening protocol as an added precaution. These criteria were used to identify adolescents requiring further assessment of disordered eating but were not considered diagnostic of an eating disorder.

Binge eating

The 16-item Binge Eating Scale (BES) was used to assess binge eating severity at baseline and week-4, -16 and -52. Statements within each question are assigned scores from 0 to 3 and summed to provide a total score from 0 to 46, with higher scores indicating more severe binge eating. A score ≤17 indicates no binge eating, scores 18 to 26 indicate mild/moderate binge eating and ≥27 indicates severe binge eating. The BES has been shown to have good psychometric properties to identify adults with obesity at risk of binge eating disorder (sensitivity 0.51-0.98, specificity 0.48-0.76 using a cut-point of 17; sensitivity 0.37-0.61, specificity 0.95-0.96 using a cut-point of 27).[4]

© 2024 Jebeile H et al. *JAMA Pediatrics*.

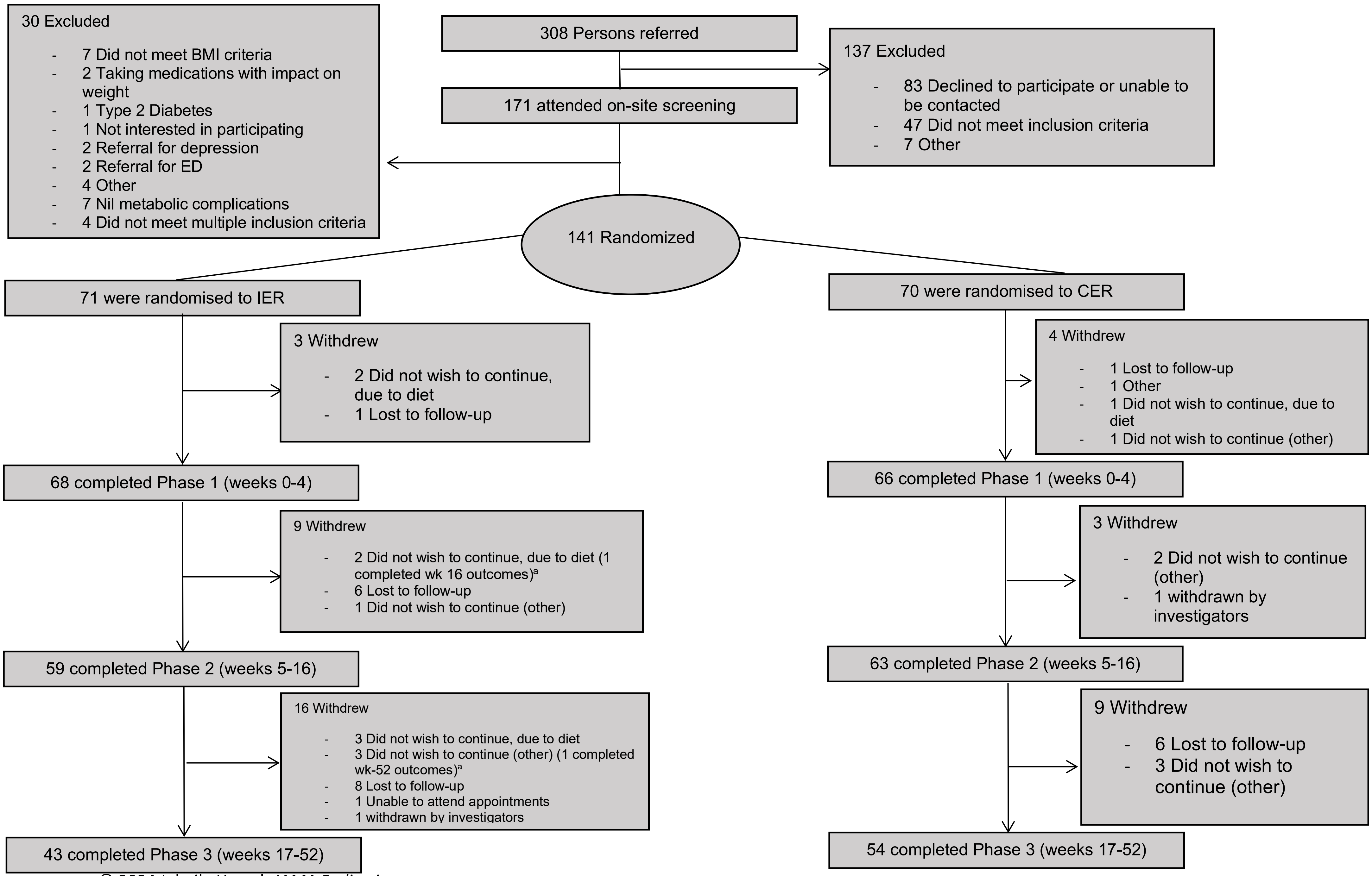

© 2024 Jebeile H et al. *JAMA Pediatrics*.

eFigure1: Consort figure

[a] Two participants withdrew from IER but returned for the next measurement visit (one at 16 weeks; one at 52 weeks)

eTable *2*: Proportion of adolescents with symptoms of depression, eating disorders and binge eating and change over time by group

	IER				CER			
**Depression**	***Baseline n=71***	***Week 4 n=65***	***Week 16 n=59***	***Week 52 n=40***	***Baseline n=70***	***Week 4 n=65***	***Week 16 n=61***	***Week 52 n=52***
No clinical significance	33 (47%)	47 (72%)	41 (70%)	28 (70%)	30 (43%)	50 (77%)	43 (71%)	32 (62%)
Subthreshold depression symptoms	21 (30%)	12 (19%)	11 (19%)	7 (18%)	26 (37%)	11 (17%)	14 (23%)	17 (33%)
Possible major depressive episode	4 (6%)	3 (5%)	1 (2%)	2 (5%)	2 (3%)	1 (2%)	1 (2%)	1 (2%)
Probable major depressive episode	5 (7%)	3 (5%)	2 (3%)	1 (3%)	1 (1%)	3 (5%)	2 (3%)	1 (2%)
Major depressive episode	8 (11%)	0	4 (7%)	2 (5%)	11 (16%)	3 (5%)	1 (1%)	1 (2%)
**Eating disorder symptoms**	***n=71***	***n=65***	***n=59***	***n=40***	***n=70***	***n=65***	***n=61***	***n=52***
No disordered eating (score <2.7, no behaviours)	17 (24%)	44 (69%)	27 (46%)	15 (38%)	13 (19%)	41 (63%)	33 (54%)	20 (39%)
Global score ≥ 2.7	27 (38%)	9 (13%)	12 (20%)	9 (23%)	26 (37%)	14 (20%)	7 (12%)	7 (14%)
Purging, ≥ 1 episode	4 (6%)	0	1 (2%)	1 (3%)	4 (6%)	1 (1%)	1 (2%)	0
Laxative use, ≥ 1 episode	1 (1%)	2 (3%)	1 (2%)	2 (5%)	2 (3%)	1 (2%)	1 (2%)	0
Overeating, ≥ 2 episodes	44 (62%)	13 (20%)	22 (37%)	23 (58%)	50 (69%)	8 (13%)	20 (33%)	24 (46%)
Loss of control, ≥ 2 episodes	29 (41%)	5 (8%)	12 (20%)	15 (38%)	29 (41%)	3 (5%)	10 (16%)	11 (21%)
Binge eating (overeating with loss of control, ≥ 2 episodes	37 (52%)	8 (13%)	20 (35%)	16 (40%)	33 (47%)	6 (9%)	15 (25%)	22 (42%)
Number of participants meeting any screening criteria	54 (76%)	20 (31%)	32 (54%)	25 (63%)	56 (80%)	24 (37%)	28 (46%)	31 (61%)
**Binge eating**	***n=68***	***n=63***	***n=59***	***n=40***	***n=67***	***n=63***	***n=60***	***n=52***
No binge eating, score ≤17	51 (75%)	56 (89%)	53 (90%)	35 (88%)	56 (84%)	58 (92%)	56 (93%)	42 (81%)
Mild/moderate BE, score 17-26	13 (19%)	5 (8%)	5 (9%)	4 (10%)	10 (15%)	4 (6%)	4 (7%)	6 (12%)
Severe BE, score ≥27	4 (6%)	2 (3%)	1 (2%)	1 (3%)	1 (2%)	1 (2%)	0	4 (8%)

Abbreviations: BES, Binge Eating Scale; CER, continuous energy restriction; CESDR-10, Centre for Epidemiological Studies Depression Scale – Revised, 10-item version for adolescents; EDE-Q, Eating Disorder Examination Questionnaire; IER, intermittent energy restriction;

See eTable 1 for categorization of CESDR-10

© 2024 Jebeile H et al. *JAMA Pediatrics*.

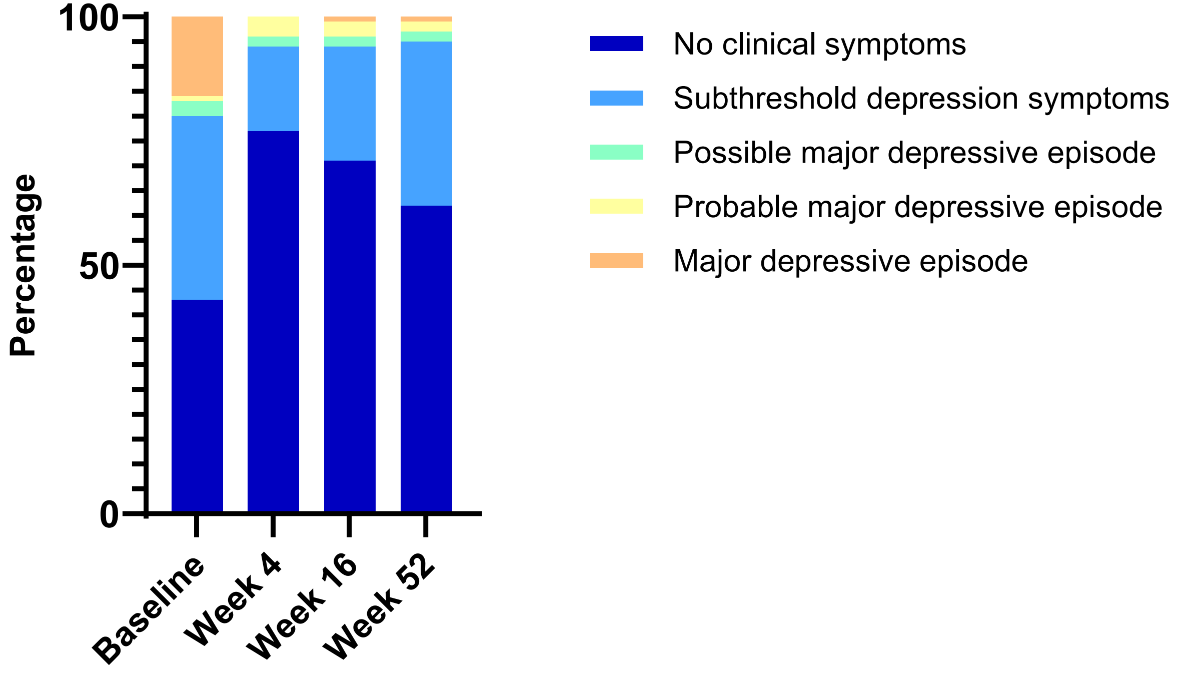

eFigure 2: Proportion of adolescents meeting depression screening criteria over time, all participants combined

As assessed by the Centre for Epidemiological Studies Depression Scale – Revised, 10-item version for adolescents; see eTable 1 for category descriptors.

© 2024 Jebeile H et al. *JAMA Pediatrics*.

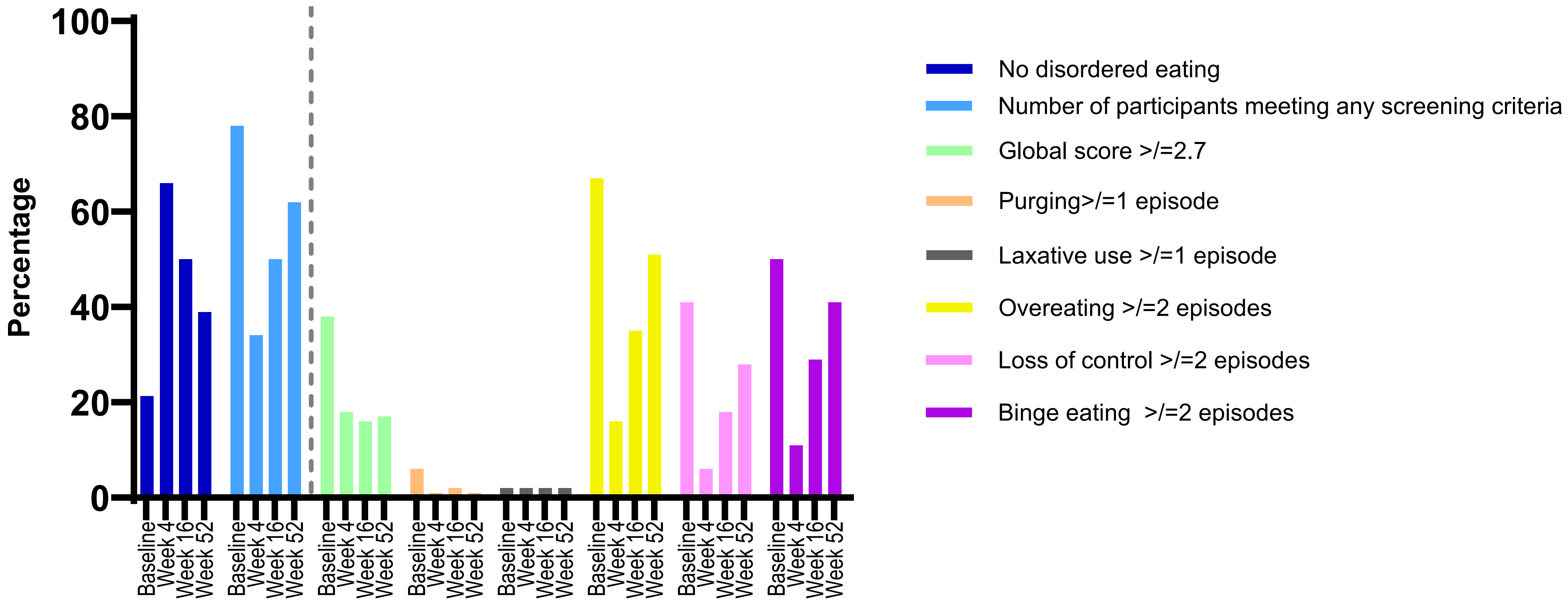

eFigure 3: Proportion of adolescents meeting eating disorder screening criteria over time, all participants combined

As measured by the Eating Disorder Examination Questionnaire.

© 2024 Jebeile H et al. *JAMA Pediatrics*.

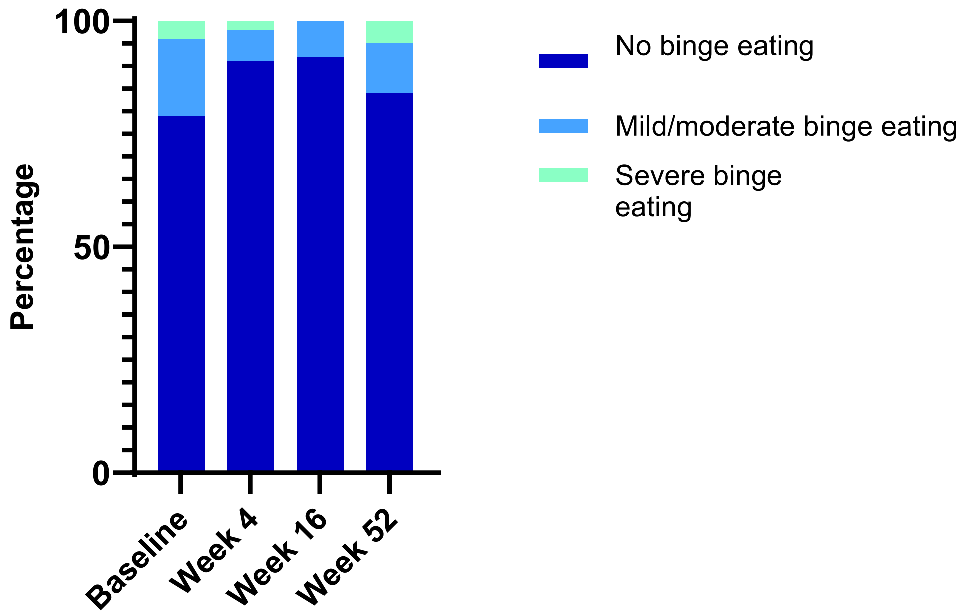

eFigure 4: Proportion of adolescents meeting Binge Eating Scale criteria over time, all participants combined

© 2024 Jebeile H et al. *JAMA Pediatrics*.

eReferences

1. Eaton WW, Smith C, Ybarra M, Muntaner C, Tien A. Center for Epidemiologic Studies Depression Scale: review and revision (CESD and CESD-R). In: Maruish ME, ed. *The use of psychological testing for treatment planning and outcomes assessment: Instruments for adults.* Lawrence Erlbaum Associates Publishers; 2004:363–377.
2. Haroz EE, Ybarra ML, Eaton WW. Psychometric evaluation of a self-report scale to measure adolescent depression: The CESDR-10 in two national adolescent samples in the United States. *J Affect Disord.* 2014;158:154-160.
3. Fairburn CG, Beglin SJ. Assessment of eating disorders: Interview or self-report questionnaire? *Int J Eat Disord.* 1994;16(4):363-370.
4. House ET, Lister NB, Seidler AL, et al. Identifying eating disorders in adolescents and adults with overweight or obesity: A systematic review of screening questionnaires. *Int J Eat Disord.* 2022;55(9):1171-1193.
5. Goldschmidt AB, Doyle AC, Wilfley DE. Assessment of binge eating in overweight youth using a questionnaire version of the Child Eating Disorder Examination with instructions. *Int J Eat Disord.* 2007;40(5):460-467.
6. Decaluwé V, Braet C. Assessment of eating disorder psychopathology in obese children and adolescents: interview versus self-report questionnaire. *Behav Res Ther* 2004;42(7):799–811.
7. Mond JM, Myers TC, Crosby RD, et al. Screening for eating disorders in primary care: EDE-Q versus SCOFF. *Behav Res Ther.* 2008;46(5):612-622.
8. Rø Ø, Reas DL, Stedal K. Eating Disorder Examination Questionnaire (EDE-Q) in Norwegian adults: Discrimination between female controls and eating disorder patients. *Eur Eat Disord Rev.* 2015;23(5):408-412.
9. Mond JM, Hay PJ, Rodgers B, Owen C, Beumont PJ. Validity of the Eating Disorder Examination Questionnaire (EDE-Q) in screening for eating disorders in community samples. *Behav Res Ther.* 2004;42(5):551-567.

© 2024 Jebeile H et al. *JAMA Pediatrics*.
