# Supplementary material for: Symptoms of Depression, Eating Disorders, and Binge Eating in Adolescents With Obesity: The Fast Track to Health Randomized Clinical Trial
Source: JAMA Pediatr. 2024 Aug 26:e242851. Online ahead of print. doi: 10.1001/jamapediatrics.2024.2851 (PMC11348093; doi:10.1001/jamapediatrics.2024.2851)
Supplement: Supplement 5. — Data Sharing Statement. [file jamapediatr-e242851-s005.pdf]

Data Sharing Statement

Jebeile. Symptoms of Depression, Eating Disorders, and Binge Eating in Adolescents With Obesity. *JAMA Pediatr*. Published August 26, 2024. doi:10.1001/jamapediatrics.2024.2851

Data
Data available: No

Additional Information
Explanation for why data not available: Data may be shared subject to further ethics approval.
